# Role of interatrial connection ablation in re-entry dynamics: an in silico evaluation

**DOI:** 10.1093/europace/euag145

**Published:** 2026-06-15

**Authors:** Patricia Martínez Díaz, Hamed Hosseini, Vladimír Sobota, Carmen Martínez Antón, Carlos López Barrera, Robin Van Den Abeele, Nele Vandersickel, Caroline Roney, Thomas Pambrun, Mélèze Hocini, Jason Bayer, Edward J Vigmond

**Affiliations:** IHU-LIRYC, L’Institut de RYthmologie et Modélisation Cardiaque, Fondation University of Bordeaux, Talence, France; Institute of Mathematics of Bordeaux, UMR 5251, University of Bordeaux, Talence, France; IHU-LIRYC, L’Institut de RYthmologie et Modélisation Cardiaque, Fondation University of Bordeaux, Talence, France; Institute of Mathematics of Bordeaux, UMR 5251, University of Bordeaux, Talence, France; IHU-LIRYC, L’Institut de RYthmologie et Modélisation Cardiaque, Fondation University of Bordeaux, Talence, France; Institute of Mathematics of Bordeaux, UMR 5251, University of Bordeaux, Talence, France; Department of Physiology, Faculty of Medicine, Masaryk University, Brno, Czech Republic; IHU-LIRYC, L’Institut de RYthmologie et Modélisation Cardiaque, Fondation University of Bordeaux, Talence, France; Institute of Mathematics of Bordeaux, UMR 5251, University of Bordeaux, Talence, France; IHU-LIRYC, L’Institut de RYthmologie et Modélisation Cardiaque, Fondation University of Bordeaux, Talence, France; Institute of Mathematics of Bordeaux, UMR 5251, University of Bordeaux, Talence, France; Biophysics Group, Department of Physics and Astronomy, Faculty of Sciences, Ghent University, Ghent, Belgium; Biophysics Group, Department of Physics and Astronomy, Faculty of Sciences, Ghent University, Ghent, Belgium; School of Engineering and Materials Science, Queen Mary University of London, London, UK; IHU-LIRYC, L’Institut de RYthmologie et Modélisation Cardiaque, Fondation University of Bordeaux, Talence, France; Hôpital Cardiologique Haut-Lévêque, CHU of Bordeaux, Pessac, France; IHU-LIRYC, L’Institut de RYthmologie et Modélisation Cardiaque, Fondation University of Bordeaux, Talence, France; Hôpital Cardiologique Haut-Lévêque, CHU of Bordeaux, Pessac, France; IHU-LIRYC, L’Institut de RYthmologie et Modélisation Cardiaque, Fondation University of Bordeaux, Talence, France; Institute of Mathematics of Bordeaux, UMR 5251, University of Bordeaux, Talence, France; IHU-LIRYC, L’Institut de RYthmologie et Modélisation Cardiaque, Fondation University of Bordeaux, Talence, France; Institute of Mathematics of Bordeaux, UMR 5251, University of Bordeaux, Talence, France

**Keywords:** Interatrial connection ablation, Computational modelling, Atrial fibrillation, Biatrial tachycardia

## Abstract

**Aims:**

Interatrial connections (IACs) may act as pathways sustaining atrial fibrillation (AF), yet their role as ablation targets remains uncertain due to challenges in identifying IAC-dependent re-entrant circuits. This study tracks re-entrant activity along IACs, characterizes IAC-dependent re-entries, and assesses the impact of in silico IAC ablation on re-entry maintenance.

**Methods and results:**

Six patient-specific biatrial bilayer models were reconstructed from CT and MRI-derived geometries, each incorporating four IACs: Bachmann’s bundle, fossa ovalis, upper posterior, and coronary sinus. The Courtemanche ionic model was used to simulate mild (M) and severe (S) AF-related electrical remodelling, with fibrosis burden ranging from 9.0% (M) to 27.6% (S). Across the 12 models, 110 sustained re-entries were induced, followed by circumferential pulmonary vein isolation. Fifteen IAC ablation strategies were tested at three different timings. Critical pathways along the six possible interatrial loops were quantified. Tachycardia cycle length (TCL) and phase singularity (PS) clusters were analysed before and after IAC ablation. Overall, 11.8% of re-entries were IAC-dependent. Larger interatrial loops were the major contributors to critical pathway formation. IAC-dependent re-entries exhibited more critical pathways, shorter TCL (194.2 vs. 201.9 ms; ΔTCL = 7.65 ms, *P* = 0.006), and more PS clusters in the RA body [8 (5–10) vs. 4 (2–6), *P* = 0.006]. Ablation timing did not influence termination rates.

**Conclusion:**

We present the first framework to track re-entrant activity along IACs. We identified IAC-dependent re-entry characteristics that may guide patient stratification and targeted ablation strategies in clinical practice.

## Graphical Abstract

**Graphical Abstract:**
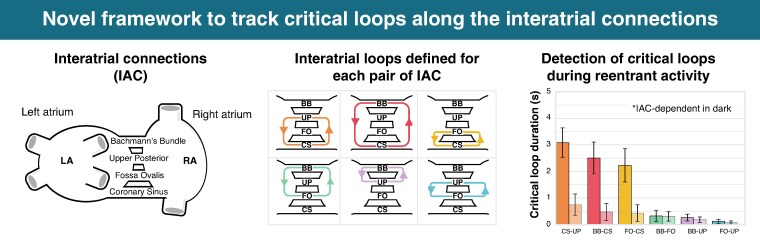

Translational perspective Patient-specific atrial computer models have been recently recognized as a future area of research in atrial fibrillation (AF) clinical guidelines. In this study, we use patient-specific computer models and a novel algorithm to track re-entrant loops along the interatrial connections (IACs). Due to the constraints associated with identifying IACs in the electrophysiological laboratory and the risks of impairing atrial physiology after ablation, computer models are an excellent tool for studying the feasibility of tracking interatrial loops and evaluating their suitability as ablation targets. The ability to clinically track critical pathways sustaining re-entry along interatrial loops could help clinicians determine in which cases IAC ablation is required, leading to more personalized and effective treatment strategies for AF.

## Introduction

The right atrium (RA) is electrically coupled to the left atrium (LA) through several muscular bundles known as the interatrial connections (IACs). During sinus rhythm, IACs ensure coordinated biatrial activation. Anatomical studies of human heart specimens have identified specific regions with a higher likelihood of IAC presence, with notable inter-individual variability in their number, size, and location.^1^ In general, four IACs have been described as major contributors to interatrial coupling: Bachmann’s bundle (BB), the fossa ovalis (FO), the upper posterior bundle (UP), and the muscular sheath of the coronary sinus (CS).^1,2^

Multiple clinical, experimental and in silico studies have suggested that IACs can act as pathways contributing to the maintenance of biatrial tachycardias (biAT) including atrial fibrillation (AF), although their exact mechanism during re-entry still remains unclear.^3–8^ Assessing the contribution of IACs to re-entry maintenance is challenging in clinical practice due to the difficulty of performing synchronized biatrial mapping.^3^ Recently, studies with high-resolution three-dimensional electroanatomical mapping (EAM) have provided insights into the role of IACs during atrial tachycardia (AT) and assessed their potential as ablation targets, in particular BB, and the CS including the vein of Marshall (VOM), an oblique vein whose ostium opens to the CS.^9–14^ Nevertheless, it remains unclear whether IACs should indeed be targeted for ablation. Ablation of the IACs is technically challenging and may impair atrial physiology, causing asynchronous contraction and delayed left atrial appendage activation, potentially increasing the risk of thromboembolism.^10^

Atrial computer models incorporating IAC anatomy offer a powerful and safer framework for tracking interatrial activity and investigate the effect of IAC ablation on arrhythmia dynamics and maintenance. Some computational studies have already evaluated the effect of ablating IACs with IAC ablation linked to re-entry termination.^5,7^ However, none of them have explicitly tracked the activity in the IACs sustaining re-entry. As a result, the mechanistic reasons why ablation of a specific IAC terminates, or fails to terminate a re-entrant episode remain poorly understood. To address this gap, we generated a cohort of patient-specific computer models and mapped activation patterns along the IACs, enabling identification of interatrial re-entrant loops and their contribution to re-entry maintenance. Specifically, we aimed to (i) simulate sustained biAT, (ii) track critical pathways along interatrial loops during re-entrant biatrial activity, (iii) identify characteristics of IAC-dependent re-entries, and (iv) evaluate different IAC ablation strategies.

## Methods

**Figure 1:**
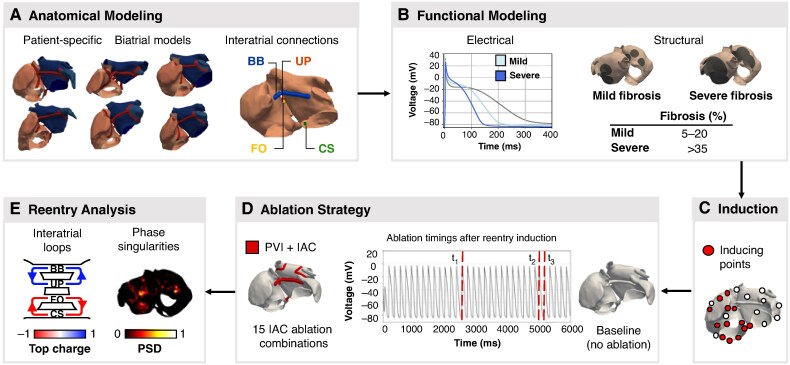
**Study methodology.**  *(A*) Six patient-specific biatrial models derived from CT and MR imaging were built including four rule-based interatrial connections. *(B*) Two levels of electrical and structural remodelling were set to study different substrate conditions (healthy action potential is shown in grey). *(C*) A decremental pacing protocol was performed to induce biatrial re-entries. *(D*) Ablation of IAC + PVI were applied simultaneously at three different timings after re-entry detection (t_1_, t_2,_ and t_3_). Fifteen IAC ablation combinations were tested. *(E*) Re-entry duration, tachycardia cycle length, number of critical interatrial pathways, and the number of phase singularities clusters were assessed. *BB, Bachmann's bundle; CS, coronary sinus; FO, fossa ovalis; IAC, interatrial connection; LA, left atrium; PVI, pulmonary vein isolation; PSD, phase singularity density; RA, right atrium; UP, upper posterior.*

The study pipeline is illustrated in *Figure 1* and further described in the following sections.

### Anatomical modelling

**Figure 2:**
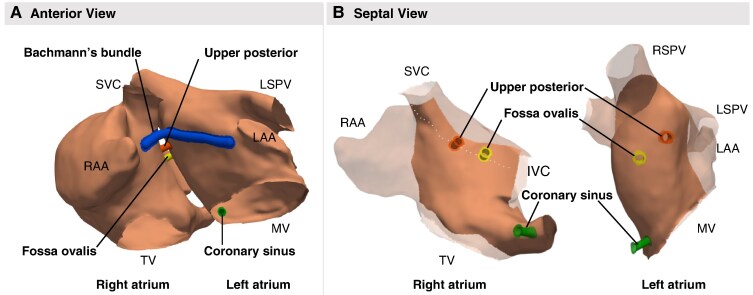
**Anatomical modelling of the interatrial connections.**  *(A*) Anterior view showing the locations of the Bachmann’s bundle (BB), upper posterior bridge (UP), fossa ovalis (FO), and CS connections. *(B*) Septal view showing the insertion locations in the right and left atrium for each connection.^19–21^ Detailed methodology available in the Supplementary material. *CS, coronary sinus; IVC, inferior vena cava; LAA, left atrial appendage; LSPV, left superior pulmonary vein; MV, mitral valve; RAA, right atrial appendage; RSPV, right superior pulmonary vein; SVC, superior vena cava; TV, tricuspid valve.*

Six patient-specific biatrial bilayer meshes, derived from clinical computer tomography and magnetic resonance imaging, were obtained as described in our previous study^15^ and downloaded from a public repository.^16^ The atrial blood pool was manually segmented,^17^ and the pulmonary veins (PVs) and valves were removed by manually placing cutting planes using ParaView 5.9.1. The endocardial surface was then extruded to form the epicardium, and coupled to form a biatrial bilayer mesh^18^ to account for atrial wall thickness using an open-source tool.^19^ The six bilayer meshes were augmented to include four IACs: BB, the FO, the UP bridge, and the CS, as illustrated in *Figure 2*. The IACs were modelled as muscular bridges (hollow tubes) connecting the epicardial surfaces of the RA and LA. The locations of the insertion sites were determined using rule-based definitions^20,21^ based on anatomical observations indicating a higher likelihood of IAC presence.^1^ The detailed methodology for IAC modelling is provided in the Supplementary material.

### Functional modelling

Atrial cellular electrophysiology was modelled using the Courtemanche–Ramirez–Nattel (CRN) mathematical model.^22^ Baseline cellular heterogeneity and anisotropic propagation in the atria were incorporated as described by Krueger *et al.*^17^ To simulate electrophysiological remodelling observed in patients with persistent AF, the maximum conductances of several ionic currents were scaled relative to the healthy CRN model.^23^ Specifically, the conductances of the following ionic channels were scaled: the L-type calcium (g_CaL_) and the sodium (g_Na_) channels; the transient outward (g_to_), the slow delayed rectifier (g_Ks_), the rapid (g_Kr_), the ultrarapid (g_Kur_), and the inward rectifier (g_K1_) potassium channels; the sarcoplasmic calcium pump current (maxI_pCa_); and the sodium-calcium exchanger (maxI_NaCa_), as summarized in Supplementary material online, *Table S1*. The applied scaling factors were: g_CaL_ × 0.45, g_to_ × 0.35, g_Ks_ × 2.0, g_Kr_ × 1.60, g_Kur_ × 0.50, g_K1_ × 2.0, maxI_pCa_ × 1.5, and maxI_NaCa_ × 1.60.^23^ The set of ionic conductance changes listed above defined the severe AF remodelling state (S). The mild state (M) was defined by linearly interpolating the scaling factors between the healthy CRN model and the S remodelling values, such that each factor corresponds to 50% of the difference between both states.^15^

Mean conduction velocity (CV) of patients with persistent AF is 1.0 m/s,^24^ so to consider the two remodelling states, we introduced a 20% reduction in CV.^15^ Tissue conductivities were tuned to yield longitudinal CVs of 1.0 and 0.8 m/s, for the M and S states, respectively. Atrial tissue-level electrophysiology was modelled using the monodomain formulation and simulations were performed with the electrophysiology simulator openCARP.^25^

Fibrosis locations were defined using a controlled seed-growing method^26^ guided by clinically reported distributions of fibrotic burden in the LA and RA corresponding to Utah stages 2 and 4.^27^ Fibrosis was distributed across multiple atrial regions, including the lateral, inferior, and posterior walls of the LA, as well as the right septal wall and posterior venous wall of the RA.^28^ For each biatrial mesh, two degrees of fibrotic remodelling were generated: Utah stage 2 representing the M, and Utah stage 4 the S states, respectively. The spatial distribution of fibrotic regions across the biatrial models ranged from 5.3% to 12.7% in the M state and from 12.8% to 42.3% in the S state. To account for the complex interplay of factors contributing to fibrosis, we modelled fibrosis using a combined approach incorporating both structural alterations, via changes in tissue conductivity, and ionic remodelling to model the effects of inflammation due to the presence of the transforming growth factor-β1 (TGF-β1). Structural remodelling was represented by applying percolation to 30% of elements within fibrotic regions, reflecting structural disruptions caused by fibrosis^29^ with a reduced tissue conductivity of σ = 0.00001 S/mm.^30^ Ionic remodelling in fibrotic regions due to the presence of the TGF-β1 was modelled as follows: g_CaL_ × 0.225, g_Na_ × 0.60, g_to_ × 0.35, g_Ks_ × 2, g_Kur_ × 0.5, maxI_pCa_ × 1.5, and maxI_NaCa_ × 1.6.^23,31^

### Simulation protocol

**Figure 3:**
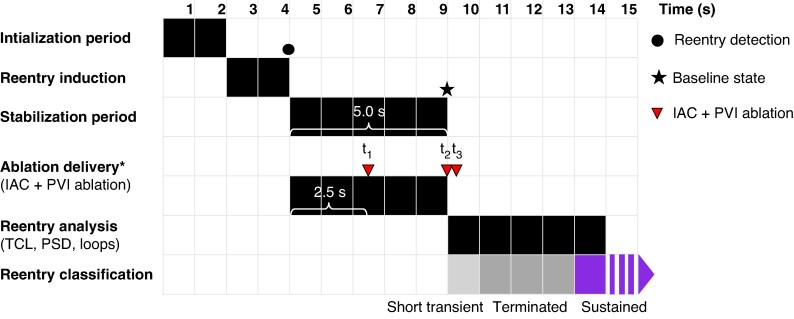
**Simulation protocol.** Biatrial models were initialized to reach a steady state. Re-entry was induced using a decremental pacing protocol. Three ablation timings (triangles) were tested: **t_1_**  **=**  **2.5 s**, **t_2_**  **=**  **5.0 s**, and **t_3_**  **=**  **5.1 s** after re-entry detection (circle).* IAC ablation + PVI were performed simultaneously and instantaneously. Re-entry analysis included TCL computation, PSD, and interatrial loop tracking. Re-entries were classified based on their duration post-ablation. The re-entry state at 5 s after re-entry detection was saved and served as the baseline state without ablation (star). Absolute simulation time is shown on top. *IAC, interatrial connection; PSD, phase singularity density; PVI, pulmonary vein isolation; TCL, tachycardia cycle length.*

The simulation protocol consists of six main stages, as shown in *Figure 3*.

### Re-entry induction

Biatrial models were initialized by pacing four times at a cycle length of 500 ms from the sinus node to reach a steady state. A decremental pacing protocol was then applied at a set of stimulation points evenly distributed across both atria, with an inter-point distance of 2 cm.^32^ Each biatrial model had 37 ± 12 stimulation points, which also served as recording locations. A re-entrant episode was considered successfully induced if a depolarization wavefront returned to the stimulation point, forming a closed-loop activation pathway,^33^ and if it persisted for at least 2 s.^32^ Induced re-entries were then continued for an additional 5 s, and the simulation state at this time was saved, which we will refer to as the baseline state (*Figure 3*). In total, 224 stimulation points were distributed across the six biatrial models. Each stimulation point was tested under two remodelling states, resulting in 448 stimulation attempts to induce re-entry. Only 110 re-entries out of 448 remained sustained for 5 s after pulmonary vein isolation (PVI), and were further included in the analysis. The distribution of re-entries in each chamber, for each remodelling state, as well as the re-entry selection criteria are provided in the Supplementary material in Supplementary material online, *Figure S2*.

### Ablation strategies

**Figure 4:**
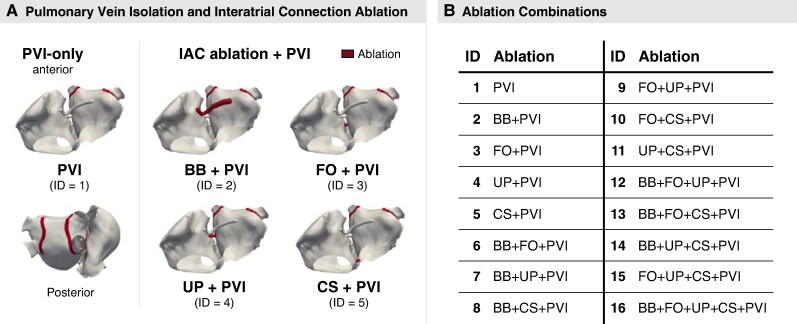
**Ablation combinations to study the role of interatrial connections on re-entry dynamics and maintenance.**  *(A*) Pulmonary vein isolation (PVI) and single interatrial connection (IAC) ablation set. (*B*) Table with the 16 ablation combinations. *BB, Bachmann’s bundle; CS, coronary sinus; FO, fossa ovalis; UP, upper posterior bridge.*

We performed IAC ablations to investigate their role in re-entry dynamics and maintenance. As PVI is the first-line treatment in catheter ablation for AF,^34^ in addition to IAC ablation, PVI lesions were placed around the PV antra following the wide antral circumferential ablation approach. IAC ablations and PVI were applied simultaneously and instantaneously. Ablation lesions were modelled as non-conductive and lesion lines were created to match the tip size of an 8 Fr (2.67 mm) ablation catheter. In total, 110 re-entries were subjected to 16 different ablation combinations including a control ablation targeting PVI-only (*Figure 4*).

### Ablation timings

Since ablation success depends on the phase and temporal stability of the simulated re-entry,^35^ we evaluated three distinct scenarios to assess the impact of ablation timing on re-entry maintenance: (i) ablation applied 2.5 s after re-entry detection (t_1_), (ii) ablation applied at 5 s (t_2_), and (iii) ablation applied with a 100 ms offset (t_3_ = t_2_ + 100 ms = 5.1 s). This offset was chosen based on half of the average cycle length observed in the baseline state simulations (200.06 ± 24.95 ms). IAC ablations were applied simultaneously. After ablation, all simulations were continued for an additional 5 s to allow classification of re-entry type based on duration. Each of the 110 re-entries was subjected to all 16 ablation combinations, resulting in 1760 simulations per ablation timing. In total, considering the three timings, and the 110 baseline state simulations, 5280 + 110 = 5390 simulations were performed.

### Re-entry analysis

Re-entries were analysed at the episode level. Specifically, a re-entry was defined as an episode of sustained activation persisting for at least 5 s after induction. Multiple simultaneous wavefronts, including rotation around anatomical obstacles, or meandering activity around excitable tissue, were considered part of a single re-entrant episode if they occurred within the same continuous activation.

Re-entries were classified based on their rhythm (AF or AT), duration and dependence on IACs. Rhythm classification of re-entries was performed before ablation (baseline state). Tachycardia cycle length (TCL) was computed by measuring the mean peak-to-peak interval of the transmembrane voltage derivative (dV/dt) at the recording locations in the biatrial meshes. In each chamber, baseline re-entries were classified as AF when either the standard deviation of the TCL was ≥20 ms or the mean TCL was <200 ms as proposed by Meyer *et al.*^36^ All other re-entries were classified as AT. Re-entries were classified according to their post-ablation duration as short-transient (<1 s), terminated (≥1 s and < 5 s), or sustained (≥5 s). Finally, re-entries were classified based on their dependence on IACs. A re-entry was considered IAC-dependent if it became short-transient or terminated after complete interatrial ablation (ablation ID = 16, corresponding to ablation of all four IACs). Otherwise, the re-entry was classified as IAC-independent. Clinically, re-entries dependent on IACs are also referred to as biAT.^36,37^

### Phase singularities

**Figure 5:**
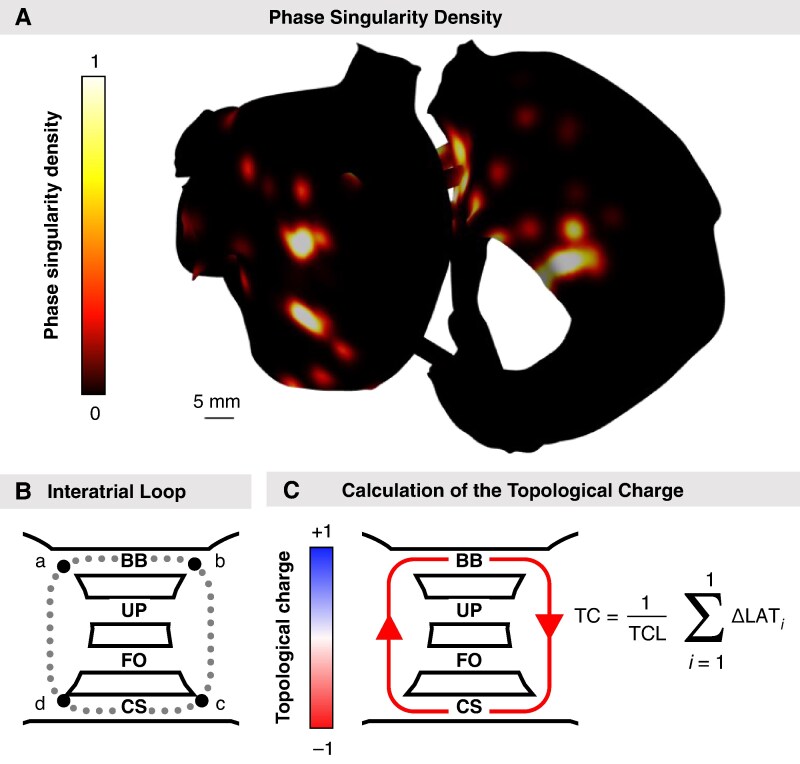
**Phase singularity density maps, definition of interatrial loops, and calculation of topological charge.**  *(A*) Phase singularity density maps were computed for each re-entry as described by Roney *et al.*^31^ (*B*) Interatrial loops were defined for each pair of IACs as closed paths connecting four manually selected vertices (a,b,c,d) by adapting the method from Van den Abeele R. *et al.*^38^ (*C)* The topological charge (TC) of a loop was calculated from the sum of the local activation times (LAT) gradient divided by the tachycardia cycle length (TCL) of the re-entry, with chirality defined by the sign of the TC. *BB, Bachmann’s bundle; CS, coronary sinus; FO, fossa ovalis; IAC, interatrial connection; UP, upper posterior bridge.*

For the baseline state re-entries, node-based phase singularity (PS) density maps (*Figure 5A*) were quantified following the method described by Roney *et al.*^31^ A detailed description of the calculation of PS can be found in the Supplementary material.

### Detection of critical pathways along interatrial loops

**Figure 6:**
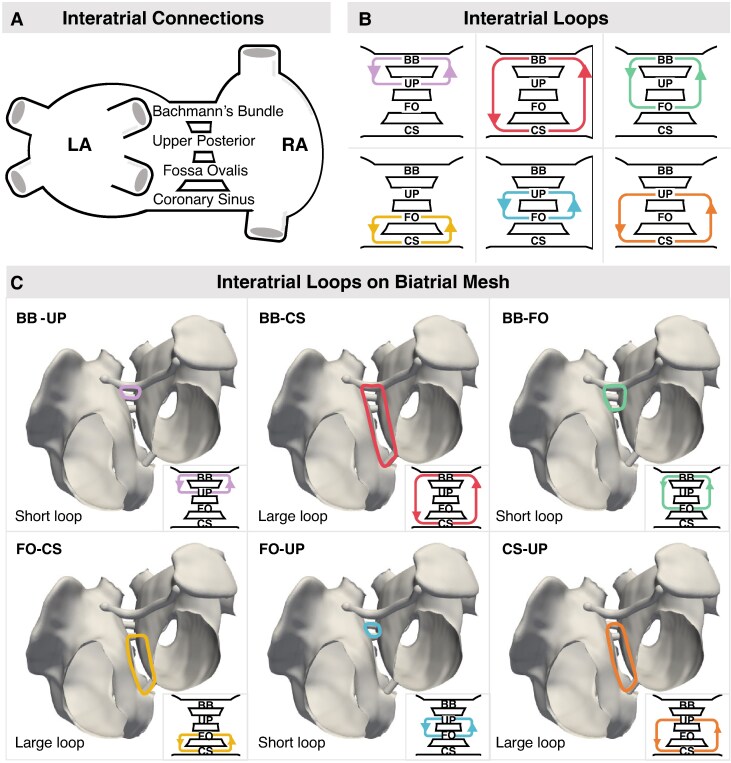
**Interatrial loops.**  *(A*) Four interatrial connections. *(B*) Schematic of the six possible interatrial loops. *(C*) Interatrial loops on a biatrial mesh. Large interatrial loops are BB-CS, FO-CS and CS-UP. *BB, Bachmann’s bundle; CS, coronary sinus; FO, fossa ovalis; IAC, interatrial connection; LA, left atrium; RA, right atrium; UP, upper posterior bridge.*

To characterize re-entrant activity involving the IACs, the method proposed by Van den Abeele *et al.*^38^ for detecting critical boundaries in the atrial body was adapted to identify critical pathways along interatrial loops. Interatrial loops were defined for each pair of IACs as closed paths connecting four manually selected vertices (*Figure 5B*), with one vertex on the right and left atrial side of each IAC. The interatrial loop was constructed by concatenating the shortest geodesic segments between these four vertices to form a continuous path. In total, six interatrial loops were defined for each biatrial mesh, as shown in *Figure 6*. A *critical pathway* was defined as an interatrial loop with a non-zero topological charge. Local activation times (LATs) were obtained by applying a −50 mV voltage threshold to the transmembrane voltage signals. LATs were transformed into phases using sawtooth mapping.^39^ The topological charge around each interatrial loop was computed as the line integral of the phase gradient divided by 2π (*Figure 5C*), corresponding to the TCL. The number of detected critical pathways was quantified over the entire re-entry duration at a sampling frequency of 100 Hz in the baseline state.

### Statistical analysis

Continuous data are presented as mean ± standard deviation. Discrete data are presented as median and interquartile range. A one-way ANOVA was performed for comparisons across more than two groups. Associations between categorical variables were assessed using the chi-square test of independence. Associations between continuous and binary categorical variables were assessed using logistic regression. Statistical significance was defined as *P* < 0.05.

### Results

#### Re-entry characteristics in the baseline state and after PVI-only

**Figure 7:**
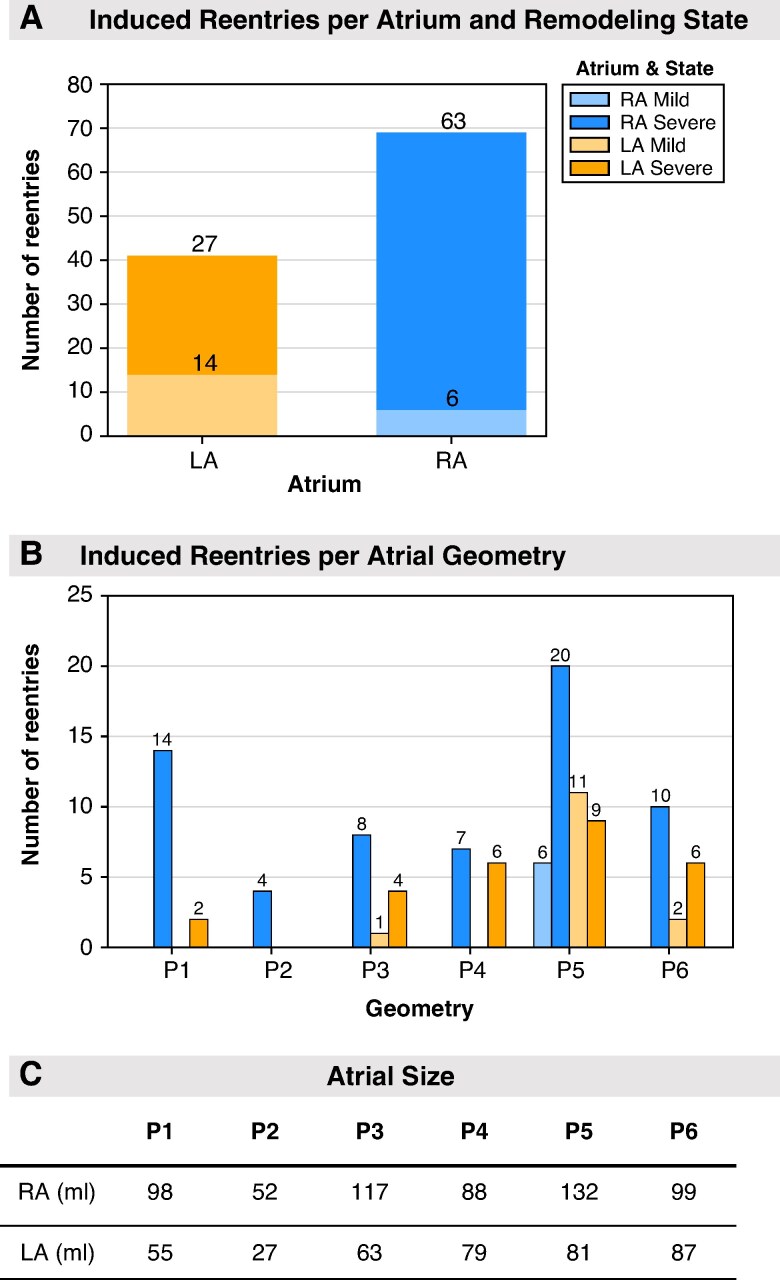
**Number of induced re-entries in the baseline state.**  *(A*) Number of re-entries per atrium and remodelling state. Re-entries induced in the right atrium (RA) are shown in blue and in the left atrium (LA) in orange. Light colour represents mild (M) remodelling and dark colour represents severe (S) remodelling. *(B*) Number of re-entries per atrial geometry. *(C*) Atrial size defined by blood volume in ml.^15,17^

**Figure 8:**
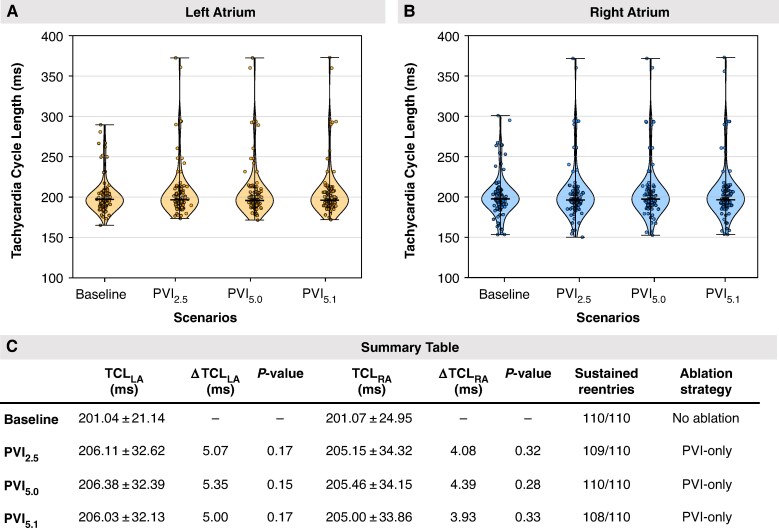
**Tachycardia cycle length (TCL) of re-entries in the baseline state and after pulmonary vein isolation (PVI).** PVI slightly increased the mean biatrial TCL compared to the baseline state. PVI was delivered at three different timings: 2.5 s, 5.0 s, and 5.1 s after re-entry detection. ΔTCL is the difference calculated considering the baseline state as reference. (*A)* Left atrium. *(B*) Right atrium. *(C*) Summary table.

Clinical characteristics of the patient cohort are provided in the Supplementary material in Supplementary material online, *Table S2*. A total of 110 re-entries were induced in the baseline state, with 69 originating in the RA and 41 in the LA. Of these, 20 occurred in the M state and 90 in the S state, as shown in *Figure 7*. The mean biatrial TCL in the baseline state was 200.06 ± 24.95 ms. All re-entries were sustained when PVI was applied at t_2_ = 5 s after re-entry detection. In general, PVI slightly increased the mean biatrial TCL compared with the baseline state (4.67 ± 0.58 ms). TCL in the baseline state and PVI cases is shown in *Figure 8*. Overall, TCL in both the RA and the LA did not significantly change when PVI was applied at different timings. At baseline, 58.2% of re-entries were classified as AF in both chambers, while 27.3% were AT, and 14.5% showed AF in at least one chamber. Following PVI, the proportion of AF in both chambers slightly decreased to 53.6% across the three ablation timings, whereas the proportion of AT in both chambers slightly increased to 30.9–33.6%. Overall, PVI shifted re-entries towards slower and more organized AT, as shown in Supplementary material online, *Figure S3*.

### Re-entry characteristics after interatrial connection ablation

Among all ablation strategies, ablation of all IACs (ablation ID = 16) produced the lowest number of sustained re-entries, whereas PVI-only (ablation ID = 1) resulted in the highest number of sustained re-entries. No significant association was observed between different ablation timings and re-entry duration classification (*P* = 0.338). Re-entry duration classification across the three ablation timings is shown in Supplementary material online, *Figure S4*. An example in which IAC ablation was ineffective in terminating re-entry, resulting in sustained activity in the RA, is shown in Supplementary material online, *Figure S5*.

**Figure 9:**
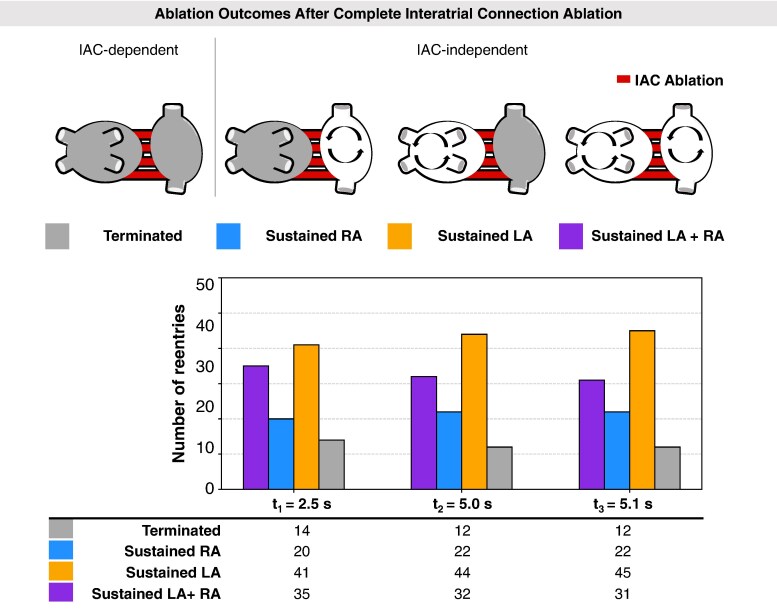
**Ablation outcomes in the three different ablation timings after complete interatrial connection (IAC) ablation.** On top, a representation of the four different outcomes after complete IAC ablation. Ablation of the four IACs delivered at t_1_ = 2.5 s, t_2_ = 5.0 s, and t_3_ = 5.1 s. Terminated = IAC-dependent. Note that short-transient re-entries were grouped into the terminated category. All re-entries terminating after complete IAC ablation were classified as IAC-dependent re-entries. *BB, Bachmann’s bundle; CS, coronary sinus; FO, fossa ovalis; UP, upper posterior bridge.*

**Figure 10:**
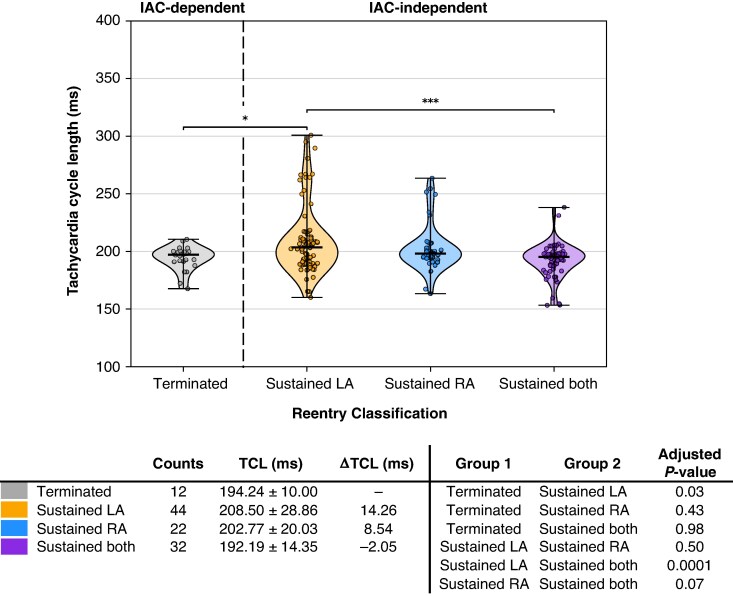
**Tachycardia cycle length (TCL) of baseline state re-entries.** Re-entries were classified based on their termination after complete interatrial connection (IAC) ablation. IAC-dependent re-entries (grey) were those terminating within 5 s after ablation delivery. IAC-dependent re-entries had a shorter TCL compared to IAC-independent re-entries. *P-value < 0.05, ***P-value < 0.0001.

Overall, 11.8% of re-entries were classified as IAC-dependent. In total, 14 re-entries were IAC-dependent at t_1_, 12 at t_2_, and 12 at t_3_ (*Figure 9*). Across the three ablation timings, nine re-entries were consistently IAC-dependent, whereas 18 re-entries were IAC-dependent in at least one timing. IAC-dependent re-entries had a shorter TCL compared to IAC-independent re-entries (194.24 ± 10.00 ms vs. 201.89 ± 0.58 ms ΔTCL = 7.65 ms, *P* = 0.006), as shown in *Figure 10*.

For the 18 IAC-dependent re-entries, we identified the minimal set of IAC ablations required to stop re-entrant activity (see Supplementary material online, *Figure S6*). In 17 out of 18 cases, ablation of a single IAC was sufficient to stop re-entry in at least one ablation timing. Of these, 13 re-entries were terminated by ablation of a specific single IAC, whereas in four re-entries, multiple single-IAC ablations were effective. Only one re-entry required ablation of a combination of two IACs. A full description of the efficacy of each ablation strategy is provided in Supplementary material online, *Figure S7* in the Supplementary material.

In 10 cases, the same IAC ablation strategy was successful across all three ablation timings, indicating time-independent termination. In contrast, the remaining eight cases showed time-dependent behaviour. Across all timings, single ablation of the CS connection (ablation ID = 5) was the most effective strategy, successfully terminating re-entry in 11 cases. Single ablation of the BB, UP, and FO connections was effective in three cases each. In particular, UP and FO ablations were generally less consistent among timings, were more sensitive to ablation timing, and more frequently appeared in combination with other IACs.

In the baseline state, the rhythm distribution of IAC-dependent re-entries in the LA and RA was 61.1% AF/AF, 33.3% AF/AT, and 5.6% AT/AT, whereas the IAC-independent group showed 57.6% AF/AF, 31.5% AT/AT, 6.5% AF/AT, and 4.3% AT/AF. A slightly higher proportion of AF/AF cases was observed in the IAC-dependent group.

Finally, we also examined the association between fibrosis in the IACs and IAC-dependent re-entries (see Supplementary material online, *Figure S8*). There was a significant negative association between IAC fibrosis and IAC-dependent re-entries (odds ratio = 0.84, *P* < 0.001), suggesting that increasing IAC fibrosis was associated with a lower likelihood of IAC-dependent re-entries.

### Detection of critical pathways along the interatrial connections

**Figure 11:**
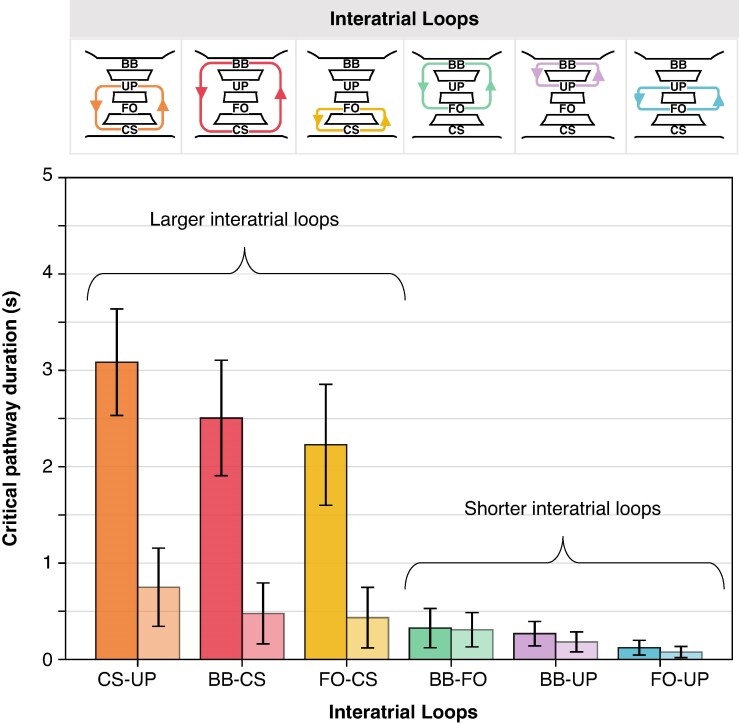
**Average duration of critical pathways along the six interatrial loops.** Critical pathways were detected throughout the entire 5 s re-entry duration in the baseline state along each interatrial loop. Darker colours represent the average duration of detected critical pathways in IAC-dependent re-entries, whereas lighter colours represent IAC-independent re-entries. *BB, Bachmann’s bundle; CS, coronary sinus; FO, fossa ovalis; IAC, interatrial connection; UP, upper posterior bridge.*

Critical pathways along the six interatrial loops were detected in the baseline state for both IAC-dependent and IAC-independent re-entries. A higher number of critical pathways was detected in larger loops, such as CS-UP, BB-CS, and FO-CS, whereas shorter loops showed fewer critical pathways in both the IAC-dependent and IAC-independent groups. In general, a greater number of critical pathways was observed in the IAC-dependent group and along loops involving the CS (*Figure 11*). An example of detected critical pathways during biAT is shown in Supplementary material online, *Figure S9* (see Supplementary material online, *Video S1*).

### Phase singularity detection

**Figure 12:**
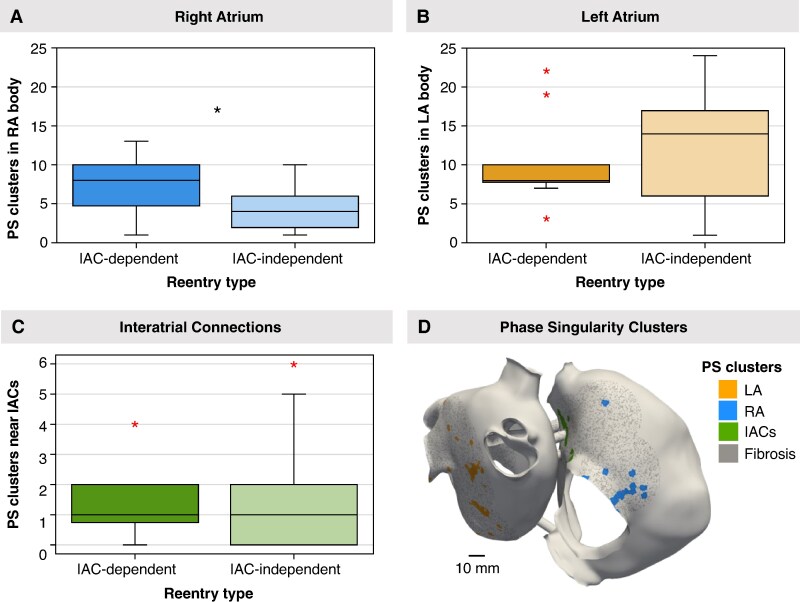
Detection of phase singularity clusters in the baseline state of re-entries dependent vs. independent to the interatrial connections. *(A*) Right atrium (RA). (*B*) Left atrium (LA). (*C*) Near the interatrial connections (IACs). (*D*) Example of spatial distribution of phase singularity (PS) clusters in a biatrial anatomy (P1) in the severe state (S). Outliers shown in red. *P-value < 0.05.

The number of PS clusters in the RA body was significantly higher in the IAC-dependent group than in the IAC-independent group [8 (5–10) vs. 4 (2–6), *P* = 0.006] (*Figure 12A*). In contrast, the number of PS clusters in the LA body did not significantly differ between the IAC-dependent and IAC-independent groups [8 (8–10) vs. 14 (6–17), *P* = 0.37] (*Figure 12B*). The number of PS clusters occurring near the IACs was comparable between IAC-dependent and IAC-independent re-entries [1 (1–2) vs. 1 (0–2), *P* = 0.96] (*Figure 12C* and *D*).

## Discussion

We present one of the first studies to track interatrial re-entrant activity along IAC loops and investigate the role of IAC ablation in re-entry maintenance using six patient-specific biatrial computer models under two remodelling levels (M and S). By evaluating 15 IAC ablation strategies, and three different ablation timings, we identified re-entry characteristics associated with IAC dependence and assessed in which cases IACs represent meaningful ablation targets. We were able to identify when IAC actively sustained re-entry and when they acted as passive pathways. Our four main findings are (i) larger interatrial loops were the major contributors to critical pathway formation, (ii) IAC-dependent re-entries exhibited shorter TCL, a higher number of critical pathways along interatrial loops, and a greater number of PS clusters in the RA body, (iii) for temporally stable re-entries, different ablation timings did not significantly affect ablation outcome, and (iv) single ablation seems sufficient to stop larger interatrial loops, particularly CS ablation.

### Mechanistic role of interatrial connections in re-entry maintenance

**Figure 13:**
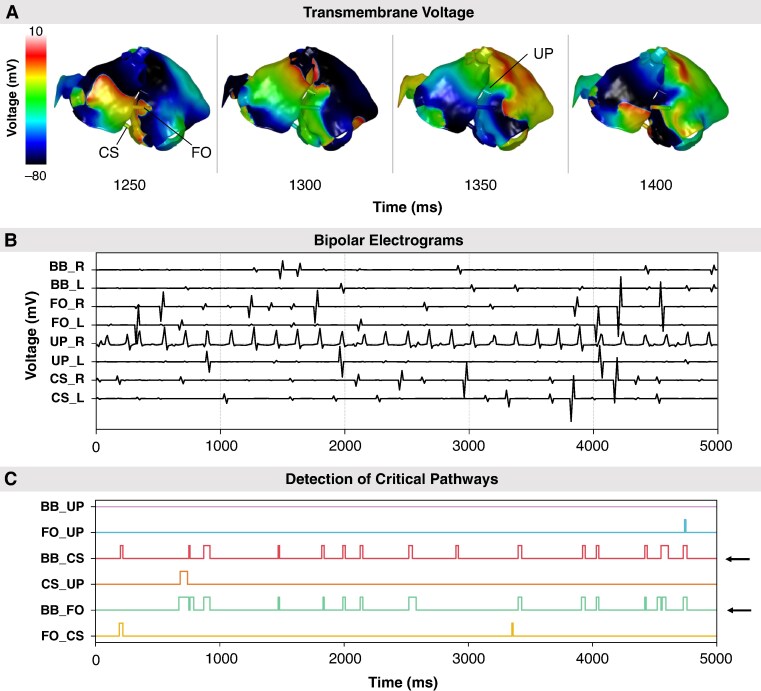
**Detection of critical pathways during atrial fibrillation.**  *(A*) Transmembrane voltage series. *(B*) Simulated bipolar electrograms (EGM) from a set of pairs of electrodes located at the right (R) and left (L) side of each interatrial connection. *(C*) Detection of critical pathways along the six interatrial loops. Arrows point out detection of critical pathways along the BB-CS and BB-FO loops. The y-axis for each interatrial loop goes from 0 (no detection) to 1 (detection). See Supplementary material online, *Video S2* in the Supplementary material.

Three main arrhythmogenic mechanisms have been proposed for IACs: (i) slow conduction leading to excitable gap formation, (ii) sites of conduction block changing IAC conduction preference, and (iii) pathways enabling wavefront propagation.^6,15,40^ Most interatrial loops reported clinically are associated with the presence of extensive prior ablation.^10,11^ However, Kitamura *et al.*^37^ proposed that re-entrant circuits may form using the interatrial septum and two IACs without the need for extensive ablation. Our study further supports that re-entrant circuits along IACs may contribute to biAT maintenance even in the absence of prior ablation lines (see Supplementary material online, *Figure S9* and Supplementary material online, *Video S1*). We also identified cases in which IACs contributed to arrhythmia maintenance during AF (*Figure 13* and Supplementary material online, *Video S2*).

To date, only activation sequences along IACs have been studied, making it challenging to determine the presence of excitable gaps in loops involving IACs. Repolarization gradients in the atria have been linked to the maintenance of re-entrant activity^41,42^ and, although not yet reported for IACs, such gradients could cause unidirectional block and promote the formation of new re-entrant pathways. Changes in IAC conduction preference may also contribute to re-entry maintenance and termination. Animal experiments have shown that, immediately before arrhythmia termination, preferential conduction through the BB can slow rapid activity and facilitate spontaneous cardioversion.^41^ A left-to-right dominant frequency gradient, in which the LA exhibits a shorter TCL than the RA, has been linked to IAC-related re-entrant activity.^7,43^ Such a gradient may promote IACs to act as passive pathways, allowing wavefront migration across chambers. Conversely, a reduction of this gradient may indicate altered interatrial coupling, for example, due to the presence of epicardial connections between the RA and the right superior PVs, thereby altering IAC conduction preference.

Clinical and in silico studies have shown that fibrotic remodelling may contribute to the development of interatrial conduction blocks.^44,45^ Delayed BB conduction may also promote shifts in preferential interatrial conduction towards more posteroinferior pathways. Advanced interatrial blocks, in which electrical conduction through the BB is blocked, are associated with the presence of AF and atrial flutter (AFl).^46^ In our study, we observed a negative association between the amount of IAC fibrosis and IAC-dependent re-entries, suggesting that advanced fibrotic remodelling may reduce the likelihood of sustaining re-entrant activity along IACs (see Supplementary material online, *Figure S6*). One possible explanation is that more advanced fibrosis within the IACs may lead to conduction failure, thereby limiting the formation of critical IAC loops required to sustain re-entry.

### Anatomical determinants of critical pathway formation along interatrial loops

Our study demonstrated that loop length and anatomical location strongly influence critical pathway formation. The three longest interatrial loops (CS-UP, BB-CS, and FO-CS) showed the highest incidence of rotational activity, whereas shorter interatrial loops (BB-FO, BB-UP, and FO-UP) rarely exhibited rotational activity. These findings suggest that larger interatrial loops are the primary contributors to critical pathway formation, while shorter interatrial loops are unlikely to contribute substantially to IAC-dependent re-entries.

Using high-density biatrial mapping, Kitamura *et al.*^37^ proposed an anatomical classification of three types of single-loop circuits that may sustain biAT. They differ according to whether the biatrial circuit involves the mitral valve, the tricuspid valve, the interatrial septum and the IACs. In our study, single loops involving IACs were also able to sustain re-entry. However, the concept that single loops act as the main drivers of AT remains controversial. Topology-based approaches for identifying re-entrant circuits in the atrial body assume a zero net topological charge (index theorem), implying that isolated single loops are theoretically unlikely.^47,48^ Reports of single-loop circuits in clinical studies may therefore reflect current limitations of biatrial mapping, potentially leading to failure to detect a second loop or incomplete rotational activity. However, the occurrence of single loops along IACs may be explained by the fact that critical pathways within IACs are not bounded to a single anatomical boundary or region, and may not be accounted for by the index theorem.^49^ Therefore, interatrial loops can appear as single loops.

### IAC-dependent re-entries exhibited shorter TCLs, a higher number of critical pathways along interatrial loops, and a greater number of PS clusters in the RA body

IAC-dependent re-entries exhibited shorter TCLs despite involving anatomical pathways. While anatomical re-entries are generally expected to present longer cycle lengths, the lower fibrosis burden in the M state likely resulted in faster conduction and consequently shorter TCLs compared with the more fibrotic S state. Although biAT has traditionally been associated with the formation of macroreentrant loops, we also identified cases in which IACs contributed to arrhythmia maintenance during AF (*Figure 13*). At baseline, 61.1% of IAC-dependent re-entries were biatrial AF. Most IAC-dependent re-entries occurred under M fibrosis, suggesting that interatrial loops may form secondary to fibrotic anchoring sites.

We observed a significantly greater number of PS clusters in the RA in IAC-dependent re-entries. Roney *et al.*^7^ reported higher mean PSD in the RA in cases of IAC ablation failure. Although they did not classify re-entries based on IAC dependence, their findings are consistent with ours, as altered RA re-entrant dynamics may better reflect the involvement of IACs in re-entry maintenance. No significant differences were observed in PS clusters within the LA body between the two re-entry groups. Finally, we did not observe significant differences in PS clusters within the interatrial septum, indicating that PS clusters near IACs alone are insufficient to distinguish IAC-dependent from IAC-independent re-entries. Importantly, PS detection based on Hilbert phase mapping may also be influenced by wave collisions, potentially leading to false-positive PS detections. Visual inspection of several simulations confirmed that the detected PSs were secondary to wave collisions around fibrotic conduction barriers and anatomical boundaries rather than sustained functional re-entries.

### Single ablation seems sufficient to stop larger interatrial loops

It remains unclear whether, to stop re-entrant loops, IACs should be ablated at breakthrough sites,^6^ or whether the entire interatrial septum should be targeted.^37^ In our study, single-IAC ablation was often sufficient to terminate re-entry; however, different re-entrant loops were observed in the same patient when re-entry was induced from multiple locations (see Supplementary material online, *Figure S5*). When analysing the efficacy of single-IAC ablation, we also observed that the CS was the most successful strategy. Therefore, we believe that the CS may represent the most feasible single IAC target. However, although single-IAC ablation may be effective to stop re-entry, it does not necessarily avoid the formation of other interatrial loops.

Ablation of IACs presents important anatomical and technical challenges that depend on the targeted structure. BB is considered the primary and largest interatrial conduction pathway, facilitating rapid activation of both atria.^1,50^ Located on the anterior aspect of the atria, the BB runs epicardially and parallel to the atrioventricular junction, extending towards both atrial appendages.^51^ BB conduction is predominantly epicardial and involves broad insertion areas which may limit ablation efficacy. Inferior muscular bundles along the CS are commonly present and provide an alternative pathway to the anterior BB connection.^2,52^ The VOM has also been implicated in arrhythmia recurrence after PVI in patients with AF, as it may serve as a common reconnection pathway.^13,14,53^ Ethanol infusion into the VOM may facilitate the formation of transmural lesions compared with radiofrequency ablation alone.^12^

While BB and CS may represent relatively accessible targets, other IACs such as the FO might be more challenging to ablate. The FO is a complex structure, with four distinct anatomical variations having been described.^54^ The FO, located within the true interatrial septum, represents a clinically important region for left atrial access.^55^ Transseptal mapping of the FO is technically challenging, and conduction at this level appears limited.^56^ The presence of net-like structures, as well as additional infoldings within the FO, may create additional pathways in the true septum, potentially limiting ablation efficacy. Bundles along the posterior interatrial groove, such as the UP and the septopulmonary bundles, have been linked to re-entry formation following incomplete roof lesion formation.^57^ Furthermore, ablating multiple IACs may disrupt physiological interatrial conduction and impair synchronized biatrial activation. Therefore, although IAC ablation may terminate re-entrant circuits, preserving adequate interatrial coupling remains an important clinical consideration.

Some studies have proposed targeting anatomical isthmuses to interrupt re-entrant circuits, such as the cavo-tricuspid isthmus (CTI), anteroseptal mitral lines or performing VOM ethanol infusion ablation.^10,11,13,14,40,53^ However, freedom from arrhythmia does not significantly differ between direct IAC ablation and anatomical ablation strategies, and the success rate for achieving durable bidirectional conduction block remains limited.^10^ A key limitation is that similar re-entrant circuits may persist by propagating through alternative IAC insertions or different epicardial bundles. Pambrun *et al.*^40^ reported relatively consistent wavefront collision sites in the LA during sinus rhythm, suggesting that improved delineation of mitral isthmus lines could avoid impairing atrial physiology. However, those studies did not track re-entrant propagation along interatrial loops. In our study, we observed cases consistent with typical AFl (see Supplementary material online, *Video S3*). Therefore, future studies could use interatrial loop tracking to more fairly compare single-IAC ablation with anatomical strategies, including VOM and CTI ablation.

Most IACs are epicardial structures, anatomically robust and resistant to radio frequency ablation. Epicardial fat may further impede the complete formation of durable transmural lesions.^36^ Even for emerging energy sources such as pulsed field ablation, which is more selective for cardiac tissue, it remains unclear whether these limitations can be fully overcome.^58^ Cryoablation appears more capable of creating transmural biatrial lesions.^59^ Nevertheless, surgical atrial lesion sets remain the only consistently effective strategy for delivering durable transmural lesions terminating macroreentrant loops.^59^ However, these surgical approaches are highly invasive and reserved for carefully selected patients, limiting their applicability in biAT.

### Different ablation timings did not affect ablation outcome in temporally stable re-entries

We ensured that all 110 re-entries remained sustained for at least 5 s after PVI to be considered truly substrate or IAC-driven. During early stages of the study, we observed that ablation timing influenced outcomes when re-entries were not intrinsically temporally stable. This observation has important implications, as ablation may appear successful in cases where the re-entry would have self-terminated, even in the absence of ablation. Previous studies investigating IAC ablation and arrhythmia maintenance have also focused on the concept of temporally stable re-entries; however, they did not explicitly assess whether re-entries were self-sustained without ablation.^5^ The concept of temporal stability becomes particularly important when ablation targets the fibrotic substrate, where re-entry reinducibility attempts could initiate temporally unstable arrhythmias.^60^ Future in silico studies of ablation strategies should account for intrinsic re-entry stability to avoid overestimating ablation efficacy.

We also assessed the effect of PVI on TCL and observed a slight increase compared with baseline. PVI has been associated with increased organization of atrial activity, mainly due to the elimination of triggering activity.^61,62^ Similarly, Coyle *et al.*^63^ also reported a reduction in the number of wavefront collisions and enhanced spatiotemporal organization after PVI. However, although slower arrhythmias are often considered less harmful, Meyer *et al.* recently reported worse survival rates in AT patients compared with AF patients. Furthermore, it is commonly assumed that slower re-entries (longer TCLs) are more likely to self-terminate compared with faster re-entries, typical of AF. In contrast, our simulations demonstrated that faster re-entries were more temporally unstable and more prone to termination. We observed that most simulated AF cases would not be sustained, which explains why, among 448 induction attempts, only 110 remained sustained for at least 5 s after PVI.

### Limitations

The limited sample size impedes broad generalization of our findings. Maintenance time was limited to 5 s for computational reasons; however, faster simulation methods^64^ could enable extended simulation times. Clinically, sustained AT is defined as a duration > 30 s. Our method still requires biatrial mapping during re-entrant activity. Most of our understanding regarding IAC structure and variability still stems from ex vivo human specimen analyses,^1^ and patient-specific assessments remain largely unavailable. We did not model personalized IAC anatomy but instead used rule-based definitions. High-resolution EAM and imaging data may enable more personalized IAC representation in atrial computer models. Our criteria for identifying IAC-dependent cases might overlook cases where the re-entrant loop changed after IAC ablation. We applied idealized lesions around the PV antra and IACs, assuming complete and permanent conduction block. In clinical practice, ablation success is influenced by several additional factors, including PV reconnection, lesion durability, tissue inflammation, progressive structural, and electrical remodelling. Finally, a systematic evaluation of different septal fibrosis distributions and their impact on interatrial conduction and re-entry formation was not performed.^65^

## Conclusion

We presented one of the first studies to track re-entrant activity along IACs to identify loops capable of sustaining re-entry and to evaluate their potential as ablation targets. The proposed framework enables the identification of IAC-dependent re-entries through the detection of critical pathways along interatrial loops. In our simulations, single-IAC ablation was sufficient to terminate IAC-dependent re-entries. We identified distinct characteristics associated with interatrial re-entrant activity that may guide patient stratification and clinical decision-making for ablation strategies. IACs may represent meaningful ablation targets in patients with lower fibrotic burden and less advanced electrical remodelling, corresponding to earlier stages of AF, in whom a higher number of interatrial loops was observed. However, further clinical and translational studies are required before systematic mapping or targeting of IAC-dependent re-entries can be considered during *de novo* ablation procedures. Our study is a step towards the clinical assessment and implementation of IAC loop mapping, which may support improved detection of biatrial re-entrant loops in clinical practice.

## Supplementary Material

euag145_Supplementary_Data

## Data Availability

The data underlying this article including bilayer models, and source code to reproduce the simulated re-entries are publicly available at: The Right Atrium Affects In Silico Arrhythmia Vulnerability In Both Atria Zenodo. 2024, https://doi.org/10.5281/zenodo.10724338. Code to perform interatrial loop analysis is open-source and available at: https://gitlab.com/opendgm/opendgm.git
