# Supplementary material for: Role of interatrial connection ablation in re-entry dynamics: an in silico evaluation
Source: Europace. 2026 Jun 15;28(6):euag145. doi: 10.1093/europace/euag145 (PMC13318161; doi:10.1093/europace/euag145)
Supplement: euag145_Supplementary_Data [file euag145_supplementary_data.zip › Supplementary_Material.pdf]

Supplementary Material

Role of Interatrial Connection Ablation in Reentry Dynamics: an in silico Evaluation

Patricia Martínez Díaz[1,2*], Hamed Hosseini[1,2], Vladimír Sobota[1,2,3], Carmen Martínez Antón[1,2], Carlos López-Barrera[1,2], Robin Van Den Abeele[4], Nele Vandersickel[4], Caroline Roney[5], Méléze Hocini[1,6], Thomas Pambrun[1,6], Jason Bayer[1,2], and Edward J. Vigmond[1,2]

1. IHU LIRYC, L'Institut de RYthmologie et modélisation Cardiaque, Fondation University of Bordeaux, Talence, France
2. Institute of Mathematics of Bordeaux, UMR 5251, University of Bordeaux, Talence, France
3. Department of Physiology, Faculty of Medicine, Masaryk University, Brno, Czech Republic
4. Biophysics Group, Department of Physics and Astronomy, Faculty of Sciences, Ghent University, Ghent, Belgium
5. School of Engineering and Materials Science, Queen Mary University of London, London, UK
6. Hôpital Cardiologique Haut-Lévêque, CHU of Bordeaux, France

* Corresponding author: patricia.martinez@ihu-liryc.fr

Modeling of the Interatrial Connections

In each of the six biatrial models, four interatrial connections (IACs) were incorporated: the Bachmann's bundle (BB), the fossa ovalis (FO), the upper posterior (UP), and the coronary sinus (CS) bridges (**Figure S1**). The IACs were modeled as muscular bridges (hollow tubes) connecting the epicardial surfaces of the right atrium (RA) and left atrium (LA). As the IACs cannot be identified from imaging data, the locations of the insertion sites were determined using rule-based definitions (20,21) based on anatomical observations indicating a higher likelihood of IAC presence (1), and incorporated using an open-source tool. (19). Auxiliary paths connecting the insertion points on the epicardial surfaces of the RA and LA aided to define IAC paths, and tubular structures were constructed along these paths extending to the nearest insertion point on the opposing atrium.

Fossa Ovalis and Upper Posterior Bridges

To find the insertion points of the FO and UP on the RA, the auxiliary path AB was defined (**Figure S1A**). The starting point A was selected as the point closest to the centroid of the superior vena cava (SVC) ring, while the endpoint B corresponded to the point nearest to the centroid of the inferior vena cava (IVC) ring. Two intermediate locations along this path, at 40% and 60% of its length, were defined as points C and D, representing the insertion points of the UP and FO, respectively. Corresponding endpoints (E and F) were then identified on the LA septum as the nearest points to C

and D. Tubular connections with a radius of 1.65 mm (21) were generated along these paths to establish septal continuity.

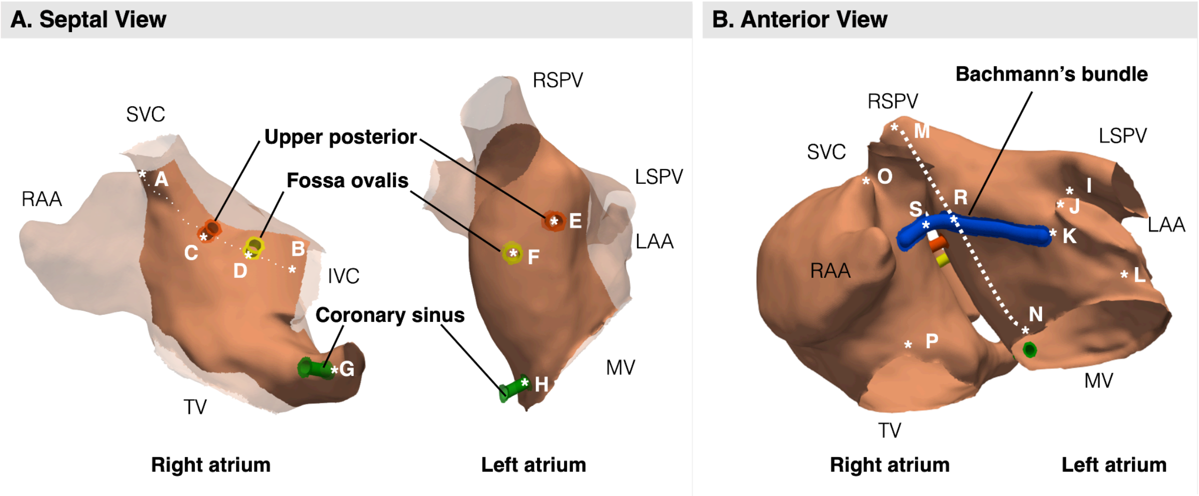

Figure S1: Modeling of the interatrial connections (IACs). Four IACs were incorporated into the bilayer models: the Bachmann's bundle (BB), the fossa ovalis (FO), the upper posterior (UP), and the coronary sinus (CS) bridges. A) Septal view. B) Anterior view. *IVC: inferior vena cava, LAA: left atrial appendage, LSPV: left superior pulmonary vein, MV: mitral valve, RAA: right atrial appendage, RSPV: right superior pulmonary vein, SVC: superior vena cava, and TV: tricuspid valve.*

Coronary Sinus Bridge

For the CS bridge, an auxiliary point was first defined by shifting the centroid of the CS ostium 10% toward the centroid of the tricuspid valve (TV) ring. The closest point to this auxiliary location on the RA epicardium was labeled G, and its corresponding nearest point on the LA surface was labeled H. A tubular structure with a radius of 1.65 mm (21) was then created along the path connecting G and H, as shown in **Figure S1A**.

Bachmann’s Bundle Bridge

Bachmann's bundle was modeled as three components: the left bundle, the intermediate bundle, and the right bundle. For the left bundle, four auxiliary points (I–L) were placed on the LA epicardial surface, extending from the left atrial appendage (LAA) to the mitral valve (MV), and connected via a geodesic path with an effective width of 2.3 mm. (21) The right bundle was defined by two points (O and P) on the RA epicardium, located near the SVC and TV, which were also connected using a geodesic path of 2.3 mm width (21).

To construct the intermediate bridge, two reference points (M and N) on the LA surface, connecting the right superior pulmonary vein (RSPV) and the MV, were used to define a path from which the midpoint (R) was determined. The closest point to R on the RA epicardium was labeled S, and a geodesic path was established between point S and R. A tubular structure with a radius of 2.13 mm (21) was then generated along this path SR.

Regional Heterogeneity and Atrial Fibrillation Modeling

Scaling factors were applied to the maximum conductances of the original Courtemanche-Ramirez-Nattel (CRN) model (22) to account for anatomical heterogeneity (**Table S1A**) and additional atrial fibrillation (AF)-induced remodeling (**Table S1B**). Specifically, the conductances of the following ionic channels were scaled: the L-type calcium (g_{CaL}), and the sodium (g_{Na}) channels; the transient outward (g_{to}), the slow delayed rectifier (g_{Ks}), the rapid (g_{Kr}), the ultrarapid (g_{Kur}), and the inward rectifier (g_{K1}) potassium channels; the sarcoplasmic calcium pump current ($maxI_{pCa}$), and the sodium-calcium exchanger ($maxI_{NaCa}$). Values shown in bold in **Table S1** indicate conductances that differ from those of healthy right atrium (RA) myocardium. AF-induced remodeling was defined by applying the following scaling factors: g_{CaL} x 0.45, g_{to} x 0.35, g_{Ks} x 2.0, g_{Kr} x 1.60, g_{Kur} x 0.50, g_{K1} x 2.0, $maxI_{pCa}$ x 1.5, and $maxI_{NaCa}$ x 1.60. The set of ionic changes listed above define the severe (S) AF remodeling state. The scaling factors for the mild state (M) state were derived by linearly interpolating the AF-induced remodeling parameters at 50%.

Table S1. Maximum scaling factors for ionic conductances used for baseline cellular heterogeneity (A) and atrial fibrillation electrical remodeling (B).

**A.**	**Regional Heterogeneity**									
**Region**	g_{CaL}	g_{Na}	g_{to}	g_{Ks}	g_{Kr}	g_{Kur}	g_{K1}	I_{pCa}	I_{NaCa}	**AR**
RA	1	1	1	1	1	1	1	1	1	3.75
PM	1	1	1	1	1	1	1	1	1	10.52
CT	**1.67**	1	1	1	1	1	1	1	1	6.56
BB	**1.67**	1	1	1	1	1	1	1	1	9
TVR	**0.67**	1	**1.53**	1	**1.53**	1	1	1	1	3.75
MVR	**0.67**	1	**2.44**	1	**1.53**	1	1	1	1	3.75
RAA	**1.06**	1	**0.68**	1	1	1	1	1	1	3.75
LAA	**1.06**	1	**0.68**	1	**1.60**	1	1	1	1	3.75
LA	1	1	1	1	**1.60**	1	1	1	1	3.75
PV	**0.75**	1	**0.75**	**1.87**	**2.40**	1	**0.67**	1	1	3.75
TGF-β1	**0.225**	**0.6**	**0.35**	**2.0**	1	**0.50**	**2.0**	**1.50**	**1.60**	-

**B.**	**Electrical Remodeling**								
**Model**	g_{CaL}	g_{Na}	g_{to}	g_{Ks}	g_{Kr}	g_{Kur}	g_{K1}	I_{pCa}	I_{NaCa}
CRN (H)	1	1	1	1	1	1	1	1	1
AF (M)	**0.73**	1	**0.68**	**1.50**	**1.30**	**0.75**	**1.50**	**1.25**	**1.30**
AF (S)	**0.45**	1	**0.35**	**2.0**	**1.60**	**0.50**	**2.0**	**1.50**	**1.60**

*AF: atrial fibrillation remodeling, AR: anisotropy ratio, BB: Bachmann's bundle, CRN: Courtemanche-Ramirez-Nattel model(22), CT: crista terminalis, H: healthy, LA: left atrium, LAA: left atrial appendage, M: mild state, MVR: mitral valve ring, RA: right atrium, RAA: right atrial appendage, PM: pectinate muscles, PV: pulmonary veins, S: severe state, TVR: tricuspid valve ring, TGF-β1: transforming growth factor beta 1. *Bold numbers indicate scaling factors differing from healthy RA/PM electrophysiology.*

Reentry Selection Criteria

In our previous study Martínez Díaz P. *et al*. (15), a pacing site was classified as inducible when reentry persisted for at least 1 s following the pacing protocol. In the present study, a stricter criterion was adopted, to improve the stability of the analyzed reentry episodes. Specifically, simulations were first extended to 2 s after pacing to determine whether reentry was sustained, followed by an additional 5 s simulation period for detailed analysis of reentry dynamics, including tachycardia cycle length, phase singularity density, and interatrial loop behavior. Consequently, transient or unstable episodes that terminated before 2 s were excluded from the analysis, resulting in a lower inducibility ratio compared to our previous publication. Out of 448 stimulation points, 197 induced reentries persisted for at least 2 s. Simulations were then extended by an additional 5 s, after which 132 reentries remained sustained. PVI was subsequently applied, and 110 reentries remained sustained thereafter.

Patient	Remodeling states (M, S)		Chamber	Stimulation points	Inducing points
P1	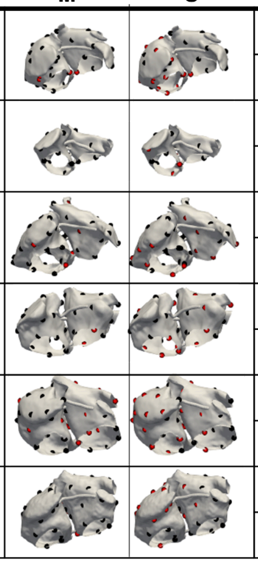	M	LA	14	2
			RA	17	3
		S	LA	14	2
			RA	17	16
P2		M	LA	7	0
			RA	10	0
		S	LA	7	0
			RA	10	4
P3		M	LA	18	3
			RA	24	3
		S	LA	18	5
			RA	24	8
P4		M	LA	18	3
			RA	19	0
		S	LA	18	6
			RA	19	9
P5		M	LA	24	13
			RA	29	6
		S	LA	24	9
			RA	29	20
P6		M	LA	23	3
			RA	21	0
		S	LA	23	7
			RA	21	10
				**448**	**197**

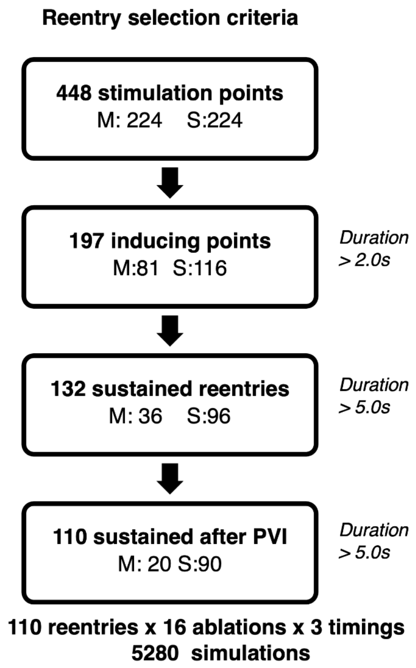

Figure S2. Reentry inducibility and selection criteria. Left: distribution of stimulation points and the number of inducing points for each chamber in each remodeling state. Right: reentry selection criteria. The main goal was to simulate reentries which would hold for 5 s after *PVI. LA: left atrium, M: mild, RA: right atrium, S: severe, and PVI: pulmonary vein isolation.*

Phase Singularity Calculation

For the baseline state reentries, node-based phase singularity (PS) density maps were quantified following the method described by Roney C. *et al.* (31) The analytic signal of the transmembrane voltage was computed using the Hilbert transform, and the instantaneous phase was extracted at each mesh node. Phase differences were then calculated between the three nodes of each triangular element. The topological charge was determined by summing the three phase differences around each triangle. PS density maps were calculated by counting the occurrence of PS lasting > 150 ms for the entire simulation time. Spatial clustering of PS regions was then performed separately for the left and right atria using the DBSCAN algorithm in Python with a distance threshold of 5 mm. Importantly, to avoid confounding effects of ablation, all PS analyses were performed in the baseline state prior to any ablation procedure. The interatrial septum was anatomically defined on the LA and RA meshes using eight manually selected landmarks and defining geodesic boundaries, following Althoff T. *et al.* (28) For each PS cluster, the centroid was computed and the minimum Euclidean distance to the septal region was measured. PS clusters located within 5 mm of the interatrial septum were classified as septal clusters.

Table S2: Clinical characteristics of subjects for the generation of biatrial models.

	P1	P2	P3	P4	P5	P6
Sex	F	F	M	M	M	M
Diagnosis	Ctl	LQT2	Ctl	LQT1	Ctl	Ctl
HR (1/min)	81	76	69	62	70	53
PWd (ms)	95	95	107	91	103	97
RA blood volume (ml)	98	52	117	88	132	99
LA blood volume (ml)	55	27	63	79	81	87
RA myocardium (mm^3)	26	12	27	38	52	21
LA myocardium (mm^3)	19	10	25	32	26	19

F: Female, M: Male, HR: Heart rate, PWd: P-wave duration, RA: right atrium, LA: left atrium, Ctl: control; LQT: long-QT syndrome, AF: atrial fibrillation. Information obtained from Martínez Díaz P. et al. 2024a. (15)

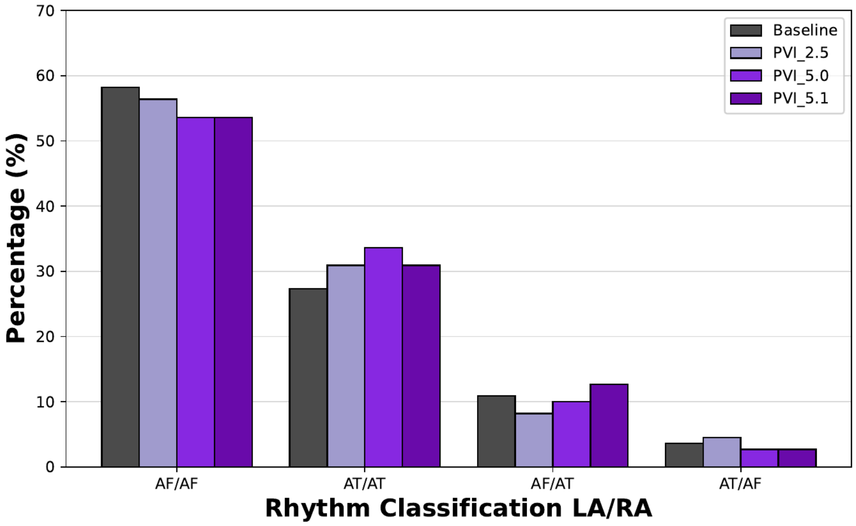

Figure S3. Rhythm classification of reentries in the baseline state and after pulmonary vein isolation (PVI). Results are presented for the left (LA) and right (RA) atrium, respectively, e.g. AT/AF stands for classification of reentry as AT in the LA and AF in the RA.

Reentry classification

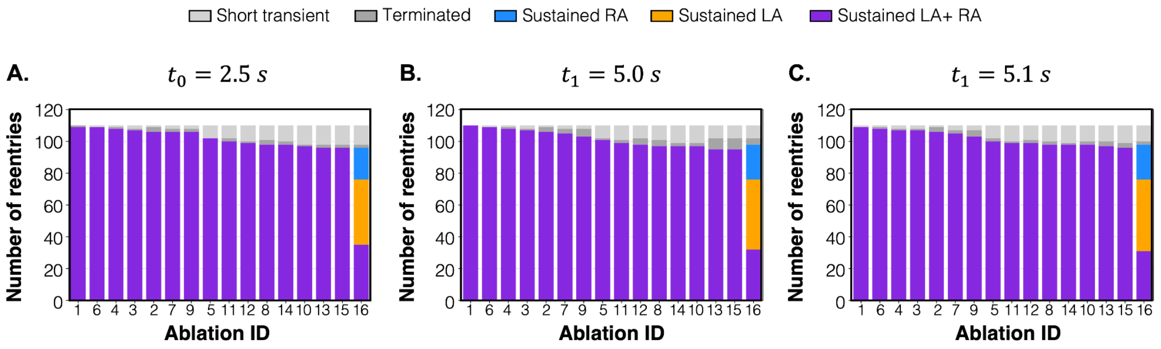

Ablation timings

	t_1= 2.5 s	t_2= 5.0 s	t_3= 5.1 s
**Short transient**	102	89	102
**Terminated**	25	46	30
**Sustained RA**	20	22	22
**Sustained LA**	41	44	45
**Sustained LA+ RA**	1572	1559	1561

Ablation combinations

ID	Ablation	ID	Ablation	ID	Ablation	ID	Ablation
**1**	PVI	**5**	CS+PVI	**9**	FO+UP+PVI	**13**	BB+FO+CS+PVI
**2**	BB+PVI	**6**	BB+FO+PVI	**10**	FO+CS+PVI	**14**	BB+UP+CS+PVI
**3**	FO+PVI	**7**	BB+UP+PVI	**11**	UP+CS+PVI	**15**	FO+UP+CS+PVI
**4**	UP+PVI	**8**	BB+CS+PVI	**12**	BB+FO+UP+PVI	**16**	BB+FO+UP+CS+PVI

Figure S4. Reentry classification in the three different ablation scenarios by ablation strategy. A. Ablation t_1= 2.5 s. B. Ablation t_2= 5.0 s. C. Ablation t_3= 5.1 s. Each of the 110 reentries was subjected to all 16 ablation combinations, leading to 1760 simulations per ablation timing. *BB: Bachmann's bundle, CS: coronary sinus, FO: fossa ovalis , IAC: interatrial connection, UP: upper posterior bridge, and PVI: pulmonary vein isolation.*

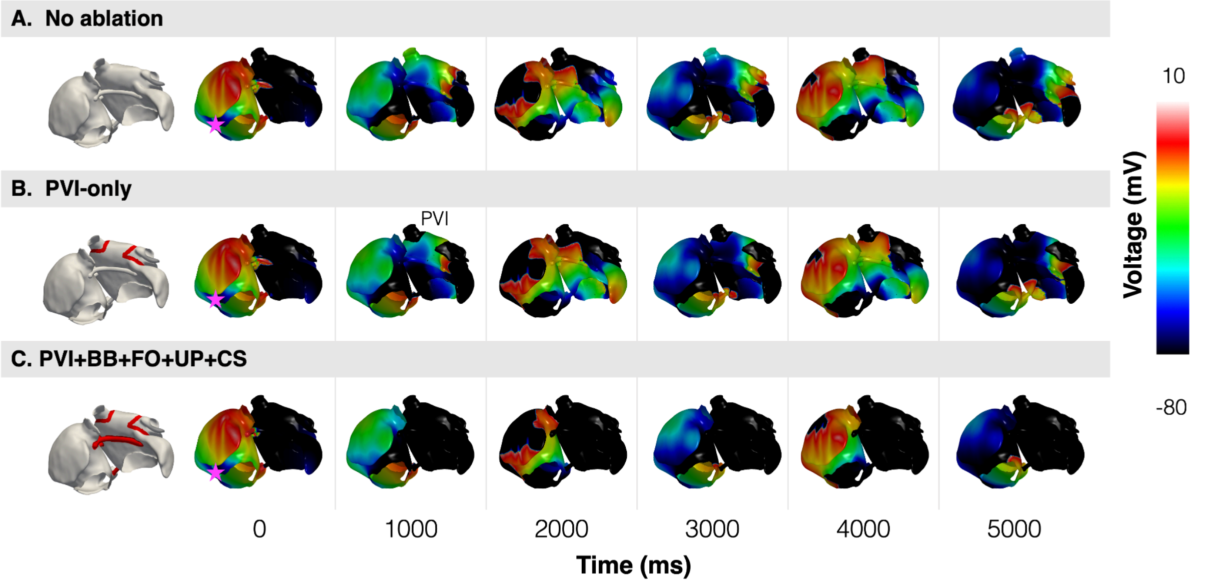

Figure S5. Example of a reentry sustained only in the right atrium and independent of the presence of interatrial connections (IACs). A. Baseline reentry without ablation. B. pulmonary vein isolation (PVI) delivered at 5 s after reentry detection. C. PVI and ablation of all IACs (ablation ID = 16) delivered at 5 s after reentry detection, corresponding to t = 0 s. The star indicates the location of the inducing point in the proximity of the right atrial appendage. IACs were not participating in the reentrant loop. *BB: Bachmann's bundle, CS: coronary sinus, FO: fossa ovalis, and UP: upper posterior bridge.*

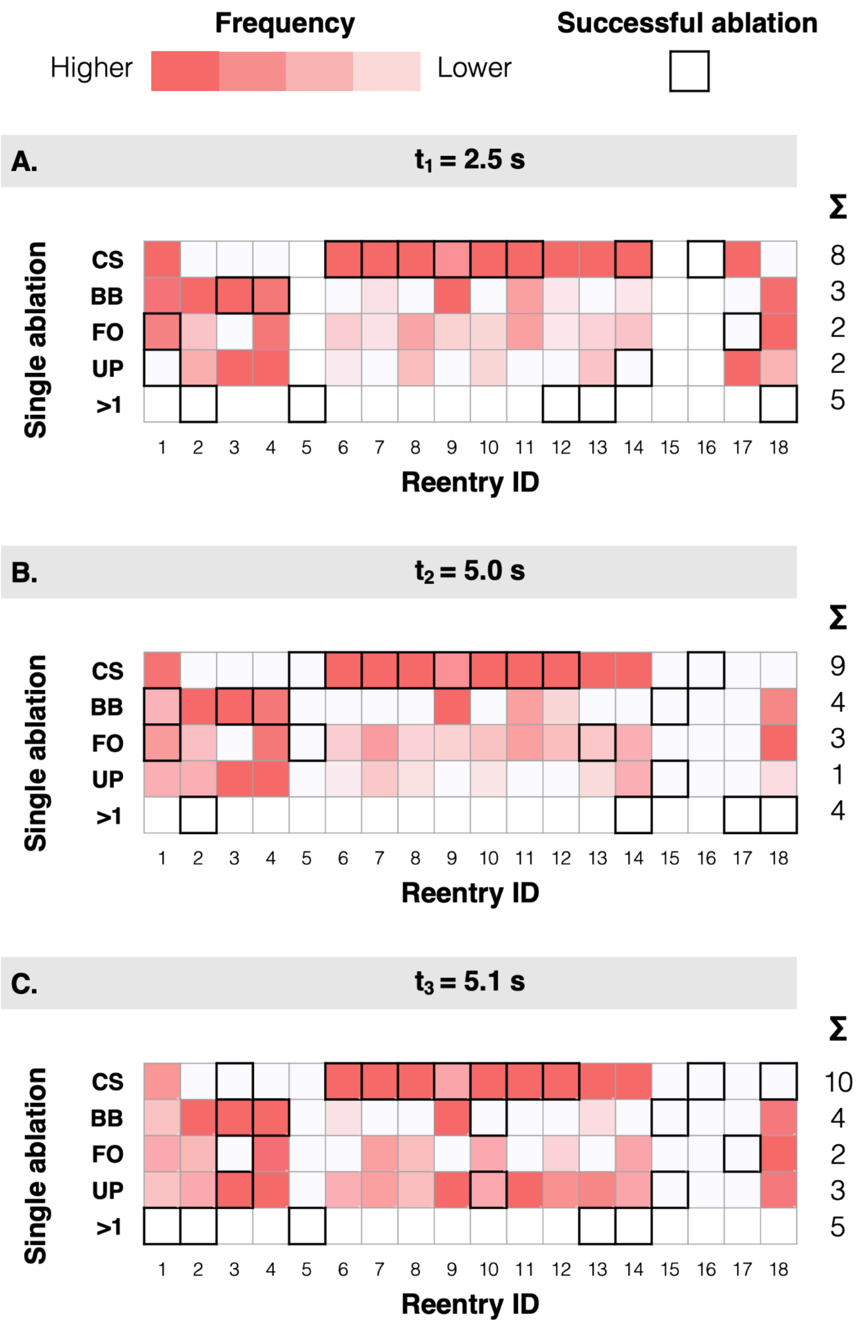

Figure S6. Efficacy of single interatrial connection (IAC) ablation across three ablation timings for IAC-dependent reentries. A. Ablation delivered at 2.5 s, B. 5.0 s and C. 5.1 s after reentry detection. Color intensity indicates the frequency with which each connection participated in detected critical pathways (Dark red = higher frequency). Cells with a black border indicate successful reentry termination following ablation of a single IAC. Each column represents the same reentry ID. >1 means that at least two IACs need to be ablated. *BB: Bachmann's bundle, CS: coronary sinus, FO: fossa ovalis , IAC: interatrial connections, and UP: upper posterior bridge.*

Ablation Combination ID

Ablation Timing: 2.5s

Reentry ID	1	6	4	9	3	7	2	5	11	13	12	10	15	8	14	16	Patient	Reentry
1	1	4	1	4	1	4	4	4	4	1	4	4	4	4	1	1	P3_bi_M_LA	point_18492_beat_2_tend_5000ms_
2	4	4	4	4	4	4	4	4	4	4	4	0	0	4	4	2	P3_bi_S_LA	point_6493_beat_1_tend_5000ms_
3	4	4	4	4	4	1	1	4	4	1	1	4	4	1	1	1	P3_bi_S_RA	point_154100_beat_1_tend_5000ms_
4	4	4	4	4	4	1	1	4	4	0	4	4	4	1	0	0	P3_bi_S_RA	point_166165_beat_1_tend_5000ms_
5	4	4	4	4	4	4	4	4	4	0	4	0	0	4	4	0	P4_bi_S_LA	point_1263_beat_3_tend_5000ms_
6	4	4	4	4	4	4	4	0	0	0	0	0	0	0	0	0	P5_bi_M_LA	point_10645_beat_2_tend_5000ms_
7	4	4	4	1	4	4	4	0	0	0	0	0	0	0	0	0	P5_bi_M_LA	point_1128_beat_2_tend_5000ms_
8	4	4	4	4	4	4	4	0	0	0	0	0	0	0	0	0	P5_bi_M_LA	point_34092_beat_3_tend_5000ms_
9	4	4	4	4	4	4	4	0	0	0	0	0	0	0	0	0	P5_bi_M_LA	point_34900_beat_2_tend_5000ms_
10	4	4	4	4	4	4	4	0	0	0	0	0	0	0	0	0	P5_bi_M_LA	point_4841_beat_2_tend_5000ms_
11	4	4	4	4	4	4	4	0	0	0	0	0	0	0	0	0	P5_bi_M_RA	point_167452_beat_2_tend_5000ms_
12	4	4	4	1	4	4	4	4	1	4	4	1	1	4	4	3	P5_bi_M_RA	point_173215_beat_3_tend_5000ms_
13	4	4	4	4	4	4	4	4	4	0	0	4	1	0	0	0	P5_bi_M_RA	point_204954_beat_2_tend_5000ms_
14	4	4	0	0	4	0	4	0	0	0	0	0	0	0	0	0	P5_bi_M_RA	point_212663_beat_2_tend_5000ms_
15	4	4	4	4	4	4	1	4	4	4	4	4	4	1	4	3	P5_bi_S_LA	point_29382_beat_2_tend_5000ms_
16	4	4	4	4	4	4	4	0	0	0	0	0	0	0	0	0	P6_bi_S_LA	point_0_beat_3_tend_5000ms_
17	4	0	4	0	0	4	4	4	4	0	0	0	0	4	4	0	P6_bi_S_LA	point_10078_beat_3_tend_5000ms_
18	4	4	4	4	4	4	4	4	4	4	4	4	4	4	4	3	P6_bi_S_RA	point_174529_beat_2_tend_5000ms_

Ablation Timing: 5.0s

Reentry ID	1	6	4	9	3	7	2	5	11	13	12	10	15	8	14	16	Patient	Reentry
1	4	4	4	4	1	1	1	4	4	4	4	4	4	4	4	2	P3_bi_M_LA	point_18492_beat_2_tend_5000ms_
2	4	4	4	4	4	4	4	4	4	4	4	0	0	4	4	2	P3_bi_S_LA	point_6493_beat_1_tend_5000ms_
3	4	4	4	4	4	1	1	4	4	1	1	4	4	1	1	1	P3_bi_S_RA	point_154100_beat_1_tend_5000ms_
4	4	4	4	4	4	1	1	4	4	1	1	0	0	1	1	1	P3_bi_S_RA	point_166165_beat_1_tend_5000ms_
5	4	4	4	4	1	4	4	0	0	4	4	4	4	1	1	4	P4_bi_S_LA	point_1263_beat_3_tend_5000ms_
6	4	4	4	4	4	4	4	0	0	0	0	0	0	0	0	0	P5_bi_M_LA	point_10645_beat_2_tend_5000ms_
7	4	4	4	0	4	4	4	0	0	0	0	0	0	0	0	0	P5_bi_M_LA	point_1128_beat_2_tend_5000ms_
8	4	4	4	4	4	4	4	0	0	0	0	0	0	0	0	0	P5_bi_M_LA	point_34092_beat_3_tend_5000ms_
9	4	4	4	4	4	4	4	0	0	0	0	0	0	0	0	0	P5_bi_M_LA	point_34900_beat_2_tend_5000ms_
10	4	4	4	4	4	4	4	1	1	1	1	4	1	1	1	1	P5_bi_M_LA	point_4841_beat_2_tend_5000ms_
11	4	4	4	4	4	4	4	0	0	0	0	0	0	0	0	0	P5_bi_M_RA	point_167452_beat_2_tend_5000ms_
12	4	4	4	4	4	4	4	0	0	0	0	0	0	0	0	0	P5_bi_M_RA	point_173215_beat_3_tend_5000ms_
13	4	4	4	1	1	4	4	4	4	4	4	0	0	1	1	3	P5_bi_M_RA	point_204954_beat_2_tend_5000ms_
14	4	4	4	4	4	4	4	4	4	4	4	4	4	4	4	2	P5_bi_M_RA	point_212663_beat_2_tend_5000ms_
15	4	0	0	0	4	0	0	4	0	0	0	4	0	0	0	0	P5_bi_S_LA	point_29382_beat_2_tend_5000ms_
16	4	4	4	4	4	4	4	0	0	0	0	0	0	0	0	0	P6_bi_S_LA	point_0_beat_3_tend_5000ms_
17	4	4	4	4	4	4	4	4	4	4	4	4	4	4	4	2	P6_bi_S_LA	point_10078_beat_3_tend_5000ms_
18	4	4	4	4	4	4	4	4	1	1	0	1	4	1	1	1	P6_bi_S_RA	point_174529_beat_2_tend_5000ms_

Ablation Timing: 5.1s

Reentry ID	1	6	4	9	3	7	2	5	11	13	12	10	15	8	14	16	Patient	Reentry
1	4	4	4	1	4	1	4	4	4	4	4	4	4	4	4	4	P3_bi_M_LA	point_18492_beat_2_tend_5000ms_
2	4	4	4	4	4	4	4	4	4	1	4	0	0	0	0	0	P3_bi_S_LA	point_6493_beat_1_tend_5000ms_
3	0	1	0	0	0	1	1	0	0	1	1	0	0	1	1	1	P3_bi_S_RA	point_154100_beat_1_tend_5000ms_
4	4	4	4	4	4	4	0	4	4	0	4	0	1	4	4	0	P3_bi_S_RA	point_166165_beat_1_tend_5000ms_
5	4	4	4	1	4	4	4	4	4	4	4	4	4	1	1	2	P4_bi_S_LA	point_1263_beat_3_tend_5000ms_
6	4	4	4	4	4	4	4	1	4	1	0	4	1	0	0	0	P5_bi_M_LA	point_10645_beat_2_tend_5000ms_
7	4	4	4	0	4	4	4	0	0	0	0	0	0	0	0	0	P5_bi_M_LA	point_1128_beat_2_tend_5000ms_
8	4	4	4	4	4	4	4	0	0	0	0	0	0	0	0	0	P5_bi_M_LA	point_34092_beat_3_tend_5000ms_
9	4	4	4	4	4	4	4	0	0	0	0	0	0	0	0	0	P5_bi_M_LA	point_34900_beat_2_tend_5000ms_
10	4	4	1	4	4	4	0	0	4	0	4	4	4	0	0	1	P5_bi_M_LA	point_4841_beat_2_tend_5000ms_
11	4	4	4	4	4	4	4	0	0	0	0	0	0	0	0	0	P5_bi_M_RA	point_167452_beat_2_tend_5000ms_
12	4	4	4	4	4	4	4	0	0	0	0	0	0	0	0	0	P5_bi_M_RA	point_173215_beat_3_tend_5000ms_
13	4	4	4	1	4	4	4	4	1	4	4	4	4	1	4	3	P5_bi_M_RA	point_204954_beat_2_tend_5000ms_
14	4	4	4	4	4	4	4	4	4	4	4	4	4	4	4	2	P5_bi_M_RA	point_212663_beat_2_tend_5000ms_
15	4	0	0	0	4	0	0	4	0	0	0	4	0	0	0	0	P5_bi_S_LA	point_29382_beat_2_tend_5000ms_
16	4	4	4	4	4	4	4	0	0	0	0	0	0	0	0	0	P6_bi_S_LA	point_0_beat_3_tend_5000ms_
17	4	4	4	4	4	4	4	4	4	4	4	4	4	4	4	2	P6_bi_S_LA	point_10078_beat_3_tend_5000ms_
18	4	4	4	4	4	4	4	0	0	4	1	4	4	4	4	2	P6_bi_S_RA	point_174529_beat_2_tend_5000ms_

Ablation Combination ID	1	6	4	9	3	7	2	5	11	13	12	10	15	8	14	16
	PVI	BB_FO	UP	FO_UP	FO	BB_UP	BB	CS	UP_CS	BB_FO	BB_FO	FO_CS	FO_UP	BB_CS	BB_UP	BB_FO_UP_CS

0	1	2	3	4
Short-transient	Terminated	Sustained LA	Sustained RA	Sustained Both

Figure S7. Efficacy of interatrial connection (IAC) ablation in the IAC-dependent group. Results are presented for the three ablation timings.

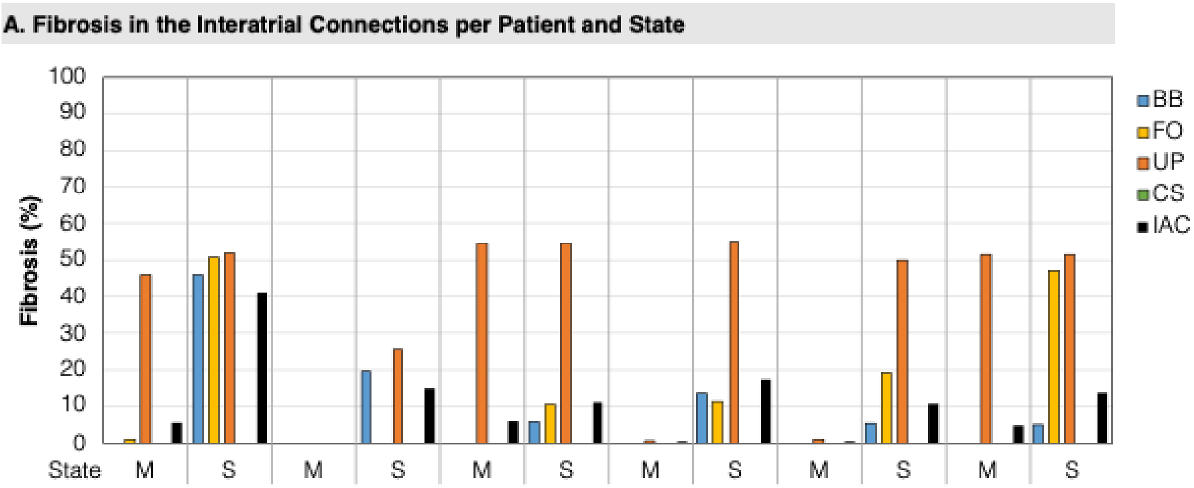

Patient	P1		P2		P3		P4		P5		P6	
State	M	S	M	S	M	S	M	S	M	S	M	S
BB	0.00	46.03	0.00	19.35	0.00	5.80	0.00	13.66	0.00	5.51	0.00	5.00
FO	1.02	50.64	0.00	0.00	0.00	10.65	0.00	11.15	0.00	19.14	0.00	46.99
UP	46.12	51.99	0.00	25.33	54.78	54.78	0.47	54.81	0.85	49.84	51.42	51.42
CS	0.00	0.00	0.00	0.00	0.00	0.00	0.00	0.00	0.00	0.00	0.00	0.00
**IAC**	5.62	41.15	0.00	15.14	5.87	11.22	0.06	17.30	0.09	10.69	4.64	13.72

Figure S8. Percentage of fibrosis in the interatrial connections for each patient. Black bar corresponds to the total amount of fibrosis in the four interatrial connections. *BB: Bachmann's bundle, CS: coronary sinus, FO: fossa ovalis , IAC: interatrial connection, M: mild state, S: severe state, and UP: upper posterior bridge.*

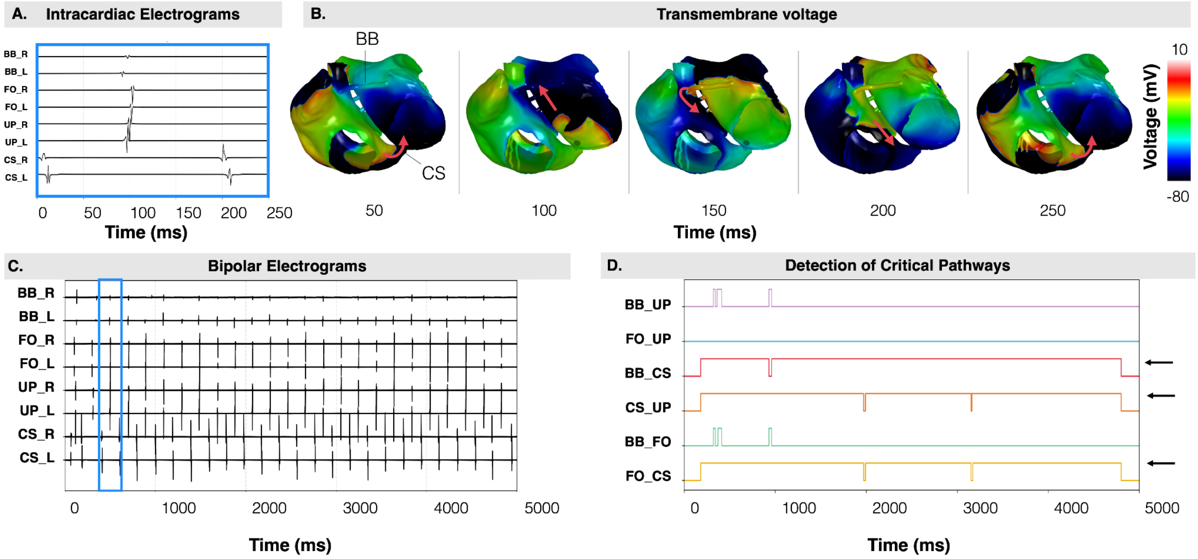

Figure S9. Example of detected critical pathways along the interatrial loop of an IAC-dependent reentry. A. Simulated bipolar electrogram (EGM) from a set of pairs of electrodes located at the right (R) and left (L) side of each interatrial connection. B. Transmembrane voltage series with red arrows showing the direction of the depolarization wavefront. C. 5000 ms EGM trace. D. Detection of critical pathways along the 6 interatrial loops, black arrows point out constant loop detection. The y-axis for each interatrial loop goes from 0 (no detection) to 1 (detection). See **Video S1** in the **Supplementary Material**. *BB: Bachmann's bundle, CS: coronary sinus, FO: fossa ovalis, IAC: interatrial connection, and UP: upper posterior bridge.*
